# Analysis of construction response for mechanized construction of metro connecting passages

**DOI:** 10.1371/journal.pone.0360916

**Published:** 2026-10-15

**Authors:** Lingbing Yi, Xiaochen Liu, Cai Yi, Peng Zhang, Lu Han

**Affiliations:** 1 China Communications Construction Company Ltd., Beijing, China; 2 Southwest Jiaotong University, Chengdu, Sichuan, China; 3 Beijing Construction Engineering Group Co. Ltd., Beijing, China; 4 China Railway Tunnel Group (Shanghai) Special High tech Co. Ltd., Shanghai, China; 5 Huanghe Jiaotong University, Jiaozuo, Henan, China; China Construction Fourth Engineering Division Corp. Ltd, CHINA

## Abstract

To investigate the influence of mechanized pipe jacking construction for connecting passages in weathered granite strata on the settlement and deformation of left and right shield sections of metro tunnels and ground surface, this paper takes the pipe jacking project of No.1 connecting passage in a typical weathered granite stratum as the research background. Numerical analysis software was used to model and analyze the whole construction process of the connecting passage using the mechanized pipe jacking method. The accuracy of the numerical model was verified by comparison with field monitoring data. On this basis, the influence laws of stratum cohesion, internal friction angle, clear distance between main tunnels, and burial depth on the deformation of the main tunnels and ground surface were systematically analyzed. The results show that: 1) The tunnel at the launching end experienced two sudden deformations during the portal breaking stage, with the bending moment increasing sharply first and then decreasing slowly; the deformation at the receiving end increased linearly with the pipe jacking advance, and reached the peak synchronously with the bending moment at the moment of portal breaking. 2) A centrally symmetrical settlement trough was formed on the ground surface, with a maximum settlement value of 2.3 mm; the maximum vertical and horizontal deformations of the main tunnel were 1.7 mm and 1.4 mm, respectively. 3) The improvement of stratum strength can reduce ground surface settlement but increase the horizontal deformation of the tunnel; the increase of the clear distance between main tunnels leads to the expansion of the settlement trough width; the increase of burial depth reduces ground surface settlement while increases the horizontal deformation of the tunnel.

## 1. Introduction

With the accelerating development of urban underground space, pipe jacking technology has become a core construction method for urban underground pipe networks, utility tunnels, metro connecting passages and other projects, owing to its advantages of low environmental disturbance and strong site adaptability [1–4]. To address the construction challenges of connecting passages in shield tunnels, Mayer et al. [1] proposed an innovative scheme for the excavation of connecting passages with segmental lining, which optimized the segment assembly and waterproof connection technology under high overburden rock pressure. Li et al. [2] revealed the deformation mechanism of the tunnel portal of cast-iron connecting passages through a three-dimensional finite element model, and clarified the mechanical control effect of lintels and bolts on the portal structure. Schwob et al. [3], relying on the Hong Kong-Zhuhai-Macao Bridge subsea tunnel project, introduced the engineering practice of constructing 41 subsea connecting passages using the pipe jacking method, providing a paradigm for the mechanized construction of connecting passages in cross-sea tunnels.

Geotechnical centrifuge model testing is an important method to reveal the stratum response induced by pipe jacking construction. Garnier et al. [5] systematically compiled the scaling laws and application boundaries of geotechnical centrifuge modelling, providing a theoretical reference for the design of relevant physical model tests. Jacking force prediction and friction reduction control are the core research contents of pipe jacking engineering. Namli and Guler [6] clarified the friction reduction effect of continuous bentonite grouting on the pipe-soil interface through laboratory tests. Ji et al. [7] established a calculation model for jacking force in granular soil strata based on the particle method, achieving accurate prediction of jacking force. Wen et al. [8] and Ma et al. [9] carried out research on the friction mechanism and jacking force prediction of rectangular pipe jacking respectively, and established calculation models adapted to rectangular sections, which greatly improved the prediction accuracy. Peerun et al. [10] systematically reviewed the core influencing factors of jacking force, and proposed a research direction to deepen the understanding of pipe-soil interaction combined with discrete element method (DEM) simulation. Yu et al. [11] proposed a modified prediction method for jacking force for curved rectangular box jacking projects in soft soil. Ma et al. [12] revealed the coupling influence law of the aspect ratio of rectangular pipes on stratum settlement and jacking force, providing a basis for the optimized design of pipe jacking sections. Sterling [4] systematically sorted out the development history and research frontiers of pipe jacking technology, and clarified the core research status of jacking force and lubrication effect. Jianbin [13] developed a complete set of key technologies for the design and manufacturing of non-circular tunnel boring machines (TBMs), expanding the engineering application of special-shaped section pipe jacking. Sheil et al. [14] proposed a dynamic prediction framework for jacking force based on Bayesian updating, solving the industry pain point that traditional methods overestimate jacking force. Wen et al. [15] established a calculation model for jacking force considering the effect of temperature for the special working condition of frozen soil strata, improving the prediction system for jacking force in special strata.

The law of stratum deformation and settlement prediction induced by pipe jacking construction are critical to ensuring the safety of surrounding buildings and structures. Ma et al. [16] clarified the zoning characteristics of stratum disturbance and the lateral influence range of pipe jacking construction through field measurement and numerical simulation. Shao et al. [17], targeting a vertical double-layer pipe jacking project in soil-rock composite stratum, revealed the superposition effect of stratum deformation caused by the construction of upper and lower pipe jacking, and modified the Peck formula to adapt to the settlement calculation of composite strata. Wang et al. [18] established an analytical model of stratum deformation for the parallel construction of rectangular pipe jacking based on Mindlin’s displacement solution and stochastic medium theory, which can reproduce the asymmetry of the settlement curve induced by construction. Ren et al. [19] derived a three-dimensional analytical solution for surface deformation induced by pipe jacking by coupling multiple construction factors, which has been verified to have good applicability through engineering projects.

The impact of multi-tunnel stacked and parallel construction on surrounding structures is a research hotspot in dense urban underground engineering. Ng et al. [20] revealed the response law and load transfer mechanism of pile group foundations induced by piggyback twin tunnel construction through centrifuge tests and numerical simulation. Soomro et al. [21,22] further studied the influence of the construction sequence of stacked twin tunnels on the settlement and stress of pile groups and single piles, and clarified the significant control effect of construction sequence on pile foundation deformation. Nasser et al. [23] analyzed the deformation impact of under-crossing tunnel construction on existing tunnels, and quantified the effect of core control parameters such as grouting. For parallel pipe jacking projects, Ma et al. [24] revealed the construction disturbance law of three parallel large-diameter pipe jacking in upper-soft and lower-hard stratum, and clarified the influence of excavation pressure and grouting pressure on surface settlement. He et al. [25] proposed a deformation control scheme for shield tunnels under-crossing existing rectangular pipe jacking tunnels, and verified the reinforcement effect of interlayer grouting combined with steel supports. Yan et al. [26] analyzed the interaction law of large-section quasi-rectangular pipe jacking side-crossing viaduct pile foundations, providing a reference for pile foundation protection in similar projects. Xu et al. [27] revealed the mechanical properties of F-type socket joints in rectangular pipe jacking tunnels through bending tests, and clarified the influence of foundation stiffness on the bearing capacity of joints.

As an important application direction of pipe jacking technology, connecting passages constructed by mechanized method have been extensively studied by domestic scholars. Ping et al. [28] compared the influence of different connecting passage opening forms on the main tunnel structure through centrifugal model tests, and clarified the control effect of opening size on tunnel deformation. Wu et al. [29] analyzed the law of grouting friction reduction effect in long-distance pipe jacking through numerical simulation, providing a basis for the optimization of grouting parameters. Jin et al. [30] developed an automatic identification and early warning system for frictional resistance in pipe jacking, realizing intelligent safety control of the construction process. Mei et al. [31], relying on a Ningbo Metro project, verified the settlement controllability of connecting passage construction by mechanized method in soft soil stratum, and clarified the distribution characteristics of its surface settlement. Zhu et al. [32] revealed the structural response law and failure process of the opening in the main tunnel of deep-buried connecting passages constructed by mechanized method through full-scale tests. Zheng et al. [33], targeting a super-large diameter shield tunnel connecting passage project, verified the structural safety control effect of special lining segments with inner steel plates. Wei et al. [34] established a full-cycle settlement displacement model for connecting passages constructed by mechanized method, and revealed the control effect of jacking force on stratum uplift and settlement. Wang et al. [35] analyzed the mechanical response of the tunnel portal structure during the hole-breaking construction of connecting passages, and proposed an optimized “ring beam reinforcement” scheme. Zhang et al. [36] revealed the mechanical characteristics of the T-junction of connecting passages, and clarified the key safety control points of bolted connections. Zhu et al. [37] clarified the evolution law of structural internal force of the main tunnel during the whole construction process of T-connection tunnels through field monitoring tests. Hu et al. [38] analyzed the influence of connecting passage excavation on the tunnel structure and surface deformation at the T-junction combined with field measurement and numerical simulation, improving the deformation prediction system for such projects.

The aforementioned studies have systematically established the theoretical framework for the mechanical response of mechanized connecting passage construction through multi-dimensional approaches, providing a solid scientific foundation for engineering optimization.However, existing studies have mostly focused on homogeneous or quasi-homogeneous strata such as soft soil, mucky soil, and seabed strata. Owing to the inherent regional heterogeneity of geotechnical engineering, soil properties vary significantly across different regions. Against this background, this paper takes the pipe jacking project of No. 1 connecting passage in the Dameisha–Xiaomeisha section of Phase II of Shenzhen Metro Line 8 as the engineering background, and uses numerical analysis software to establish a model for analyzing the entire construction process of the connecting passage with the mechanized pipe jacking method in typical weathered granite strata. The ground surface settlement, tunnel deformation, and internal force variations induced by the construction are investigated, and the accuracy of the numerical model is verified through comparison with field monitoring data. Subsequently, by adjusting the cohesion and internal friction angle of the stratum where the connecting passage is located, the clear distance between the two main line tunnels, and the burial depth of the main line tunnels, this study adopts ground surface settlement and main line tunnel deformation as the evaluation indexes to investigate and summarize the variation laws of the mechanical response of mechanical pipe jacking construction for connecting passages under different construction parameter conditions, with the aim of providing technical reference for similar engineering projects.

## 2. Overview of project

The tunnel of the Dameisha Station-Xiaomeisha Station section adopts a single-circle cross-section, and the main line tunnel is constructed by shield method with segment lining. The lining has an inner diameter of 5.5 m and an outer diameter of 6.3 m, and adopts staggered assembly of precast reinforced concrete segments. No.1 connecting passage is set at the section DK58 + 360.00 of the interval, with a burial depth of about 25.00 m and a line spacing of 18.5 m. The connecting passage is constructed by mechanized pipe jacking method, and adopts concrete segment and steel segment lining. The ordinary segment lining has a thickness of 0.25 m, an outer diameter of 3.26 m, and a ring width of 0.9 m. The geological conditions crossed by the connecting passage are completely weathered granite and sandy strongly weathered granite, and the stratum profile of the connecting passage is shown in Fig 1.

**Fig 1:**
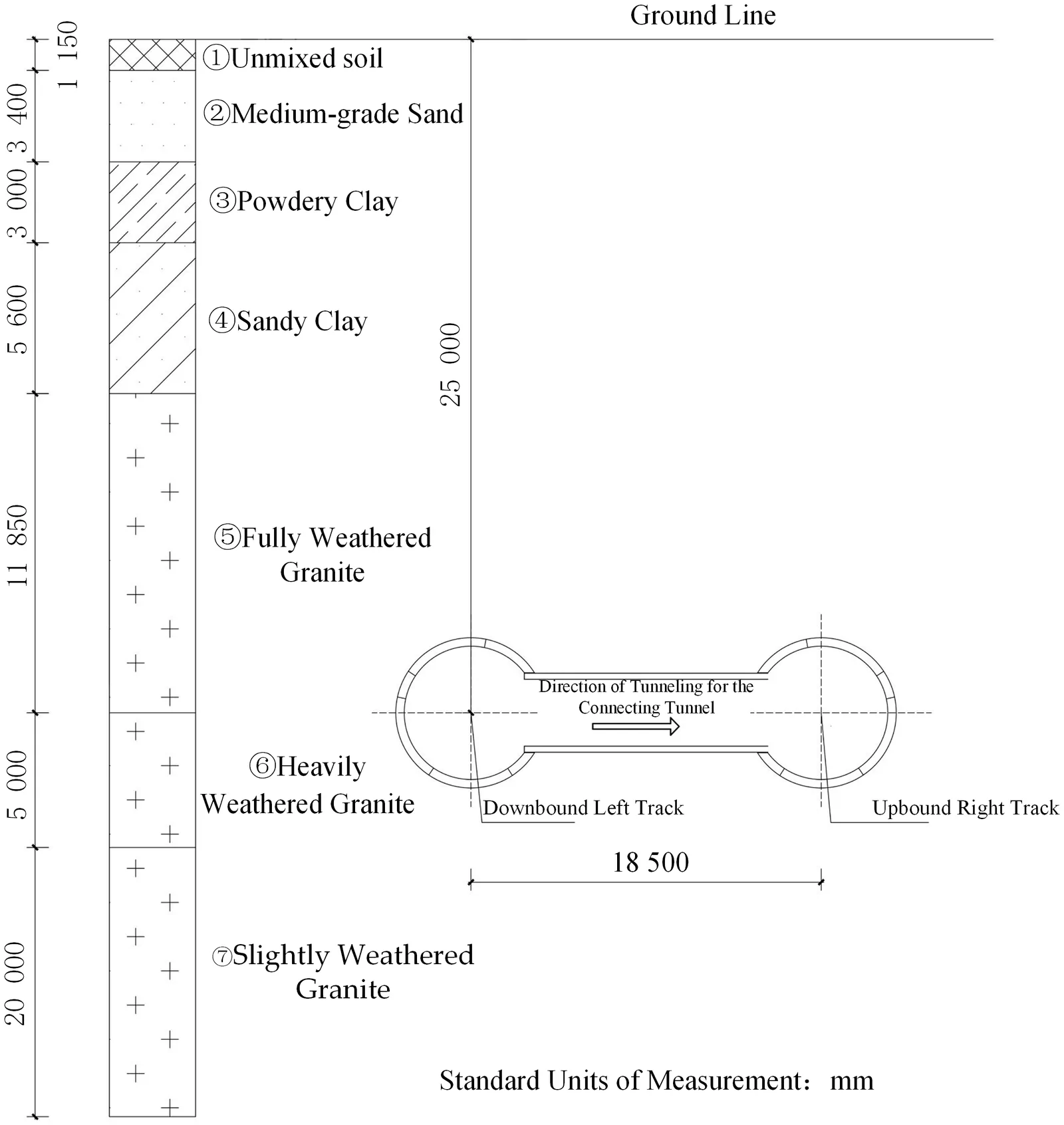
Stratum profile of the connecting passage.

## 3. Numerical calculation and analysis

In this paper, numerical analysis software was used to model and analyze the whole construction process of the connecting passage by mechanized pipe jacking method. The overall dimensions of the model are 100 m (x-axis direction) × 60 m (y-axis direction) × 80 m (z-axis direction). The top surface of the model is set as a free surface, the bottom surface restricts displacement in the vertical direction, and the remaining four sides restrict displacement in the horizontal direction. The Mohr-Coulomb constitutive model was used to simulate the soil mass, and the linear elastic constitutive model was used to simulate the structures (the left and right main tunnels and the connecting passage segments). The numerical calculation model is shown in Fig 2, and the mechanical parameters of the materials are detailed in Table 1.

**Table 1:** Mechanical property parameters of materials.

Material	Unit Weight /(kN·m ^-3^)	Modulus of Elasticity/MPa	Poisson’s Ratio	Cohesion/kPa	Internal Friction Angle/(°)
Unmixed soil	18.0	10.0	0.3	15.0	10.0
Medium-grade Sand	19.5	22.0	0.3	2.0	25.0
Powdery Clay	19.3	25.0	0.3	22.0	20.0
Sandy Clay	18.7	30.0	0.3	25.0	23.5
Fully Weathered Granite	19.2	48.0	0.3	25.0	30.0
Heavily Weathered Granite	21.0	60.0	0.3	35.0	35.0
Slightly Weathered Granite	27.0	80.0	0.3	3500.0	45.0
Ordinary Lining Segment	25.0	34.5 × 10³	0.2	–	–
Steel-Concrete Composite Segment	32.0	6.0 × 10⁴	0.2	–	–
Glass Fiber Reinforced Concrete	24.0	32.5 × 10³	0.2	–	–

**Fig 2:**
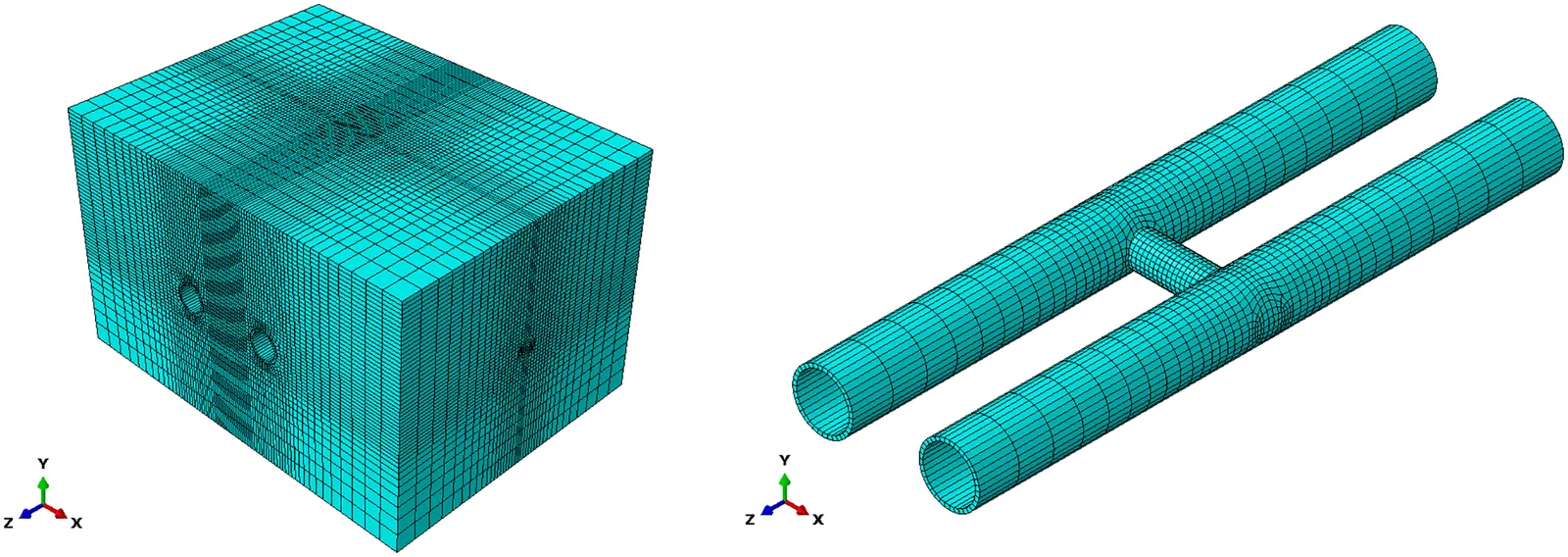
Numerical calculation model.

When simulating the whole construction process numerically, the whole construction process was divided into six construction stages with a total of 20 analysis steps.

Working Condition 1 (Step1): Initial state: Construction of the left and right line tunnels of the shield interval is completed.

Working Condition 2 (Step2): Assembly and support of the launching vehicle and pipe jacking unit are completed, ready for pipe jacking operation.

Working Condition 3 (Step3): Mechanical pipe jacking construction, launching portal breaking.

Working Condition 4 (Step16): Mechanical pipe jacking construction to the receiving portal, receiving portal breaking.

Working Condition 5 (Step19): Mechanical pipe jacking to the steel sleeve at the receiving end, construction completed.

Working Condition 6 (Step20): Disassembly of the operation trolley is completed.

### 3.1. Ground surface settlement

According to the numerical calculation results, the ground surface settlement curves are shown in Figs 3–6 below.

**Fig 3:**
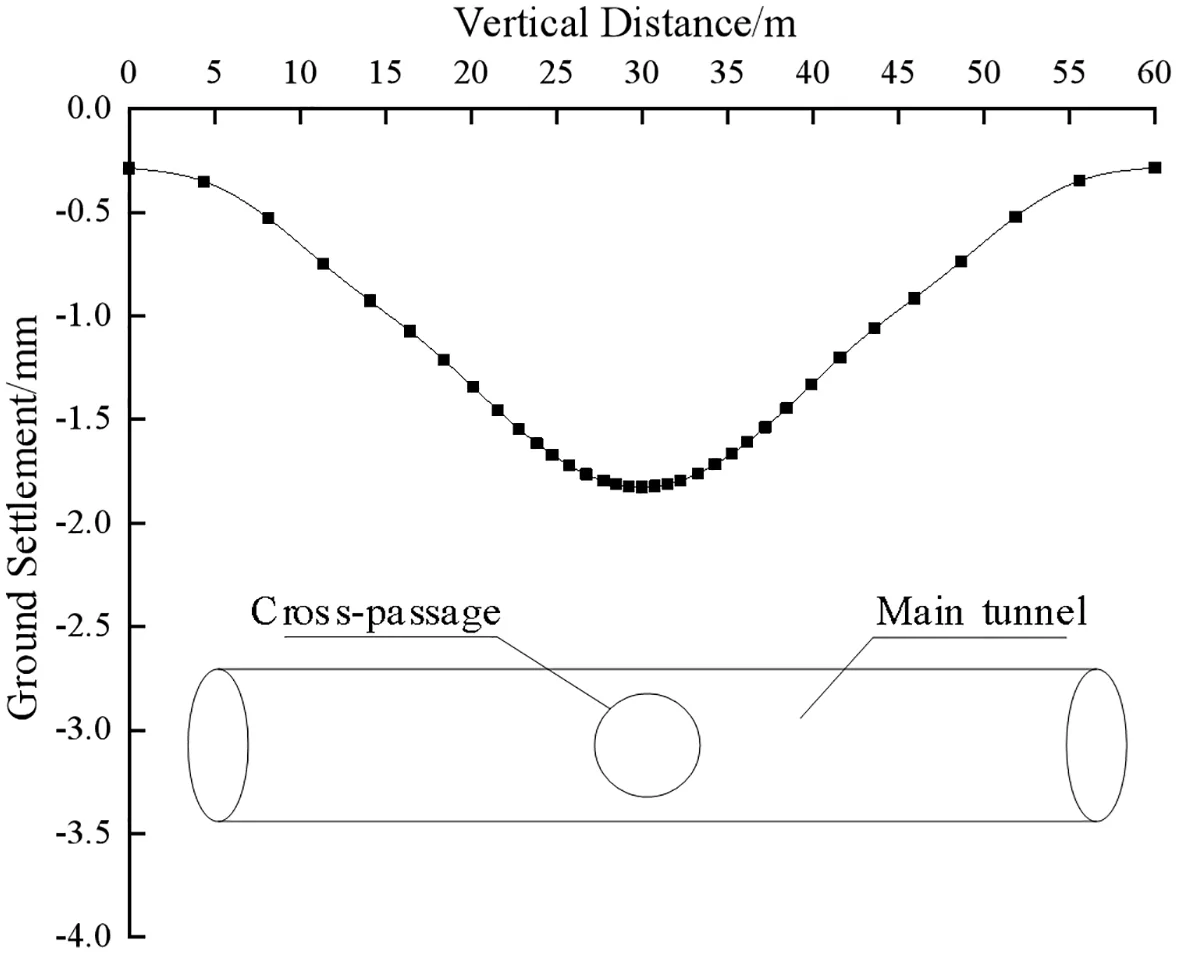
Longitudinal direction of the main tunnel at the launching end.

**Fig 4:**
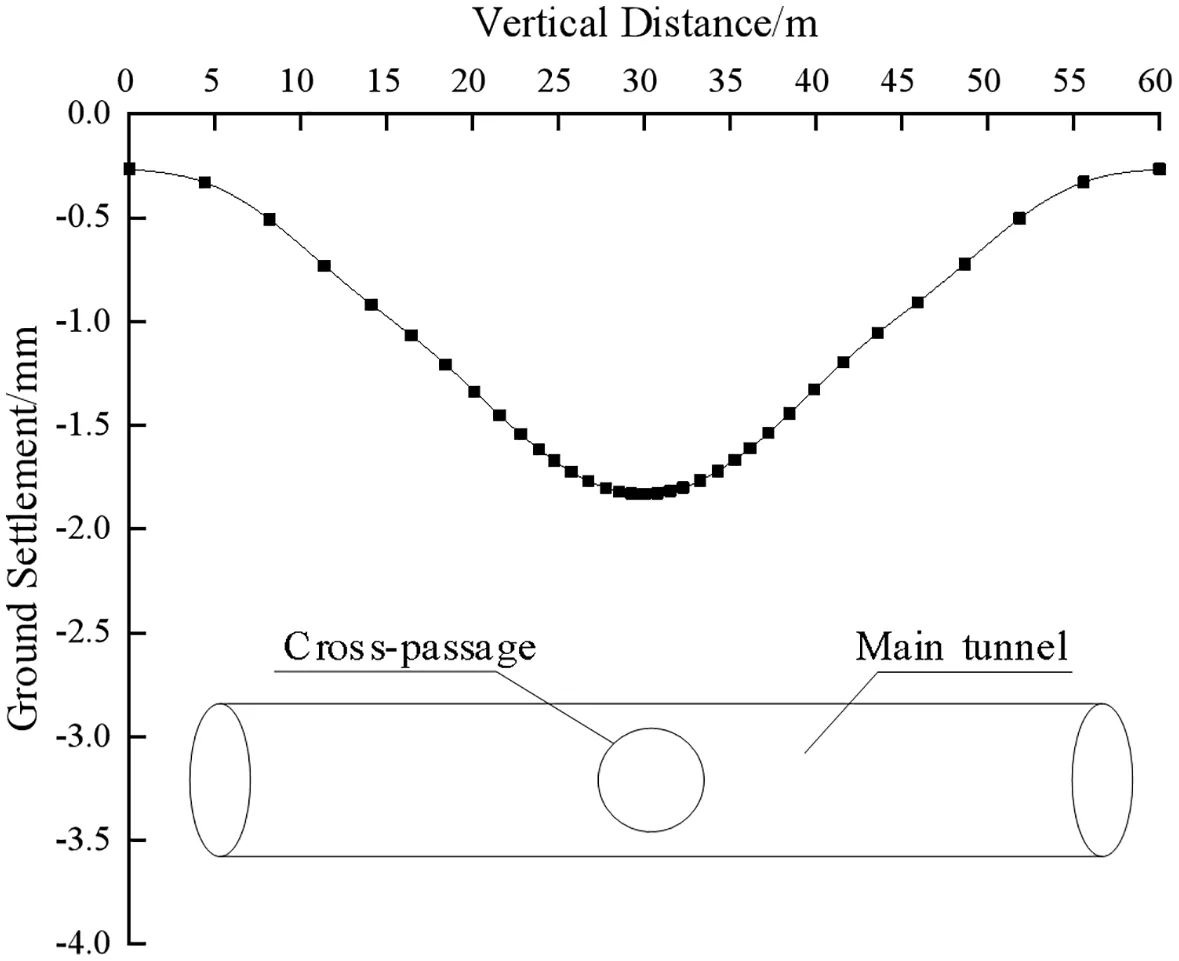
Longitudinal direction of the main tunnel at the receiving end.

**Fig 5:**
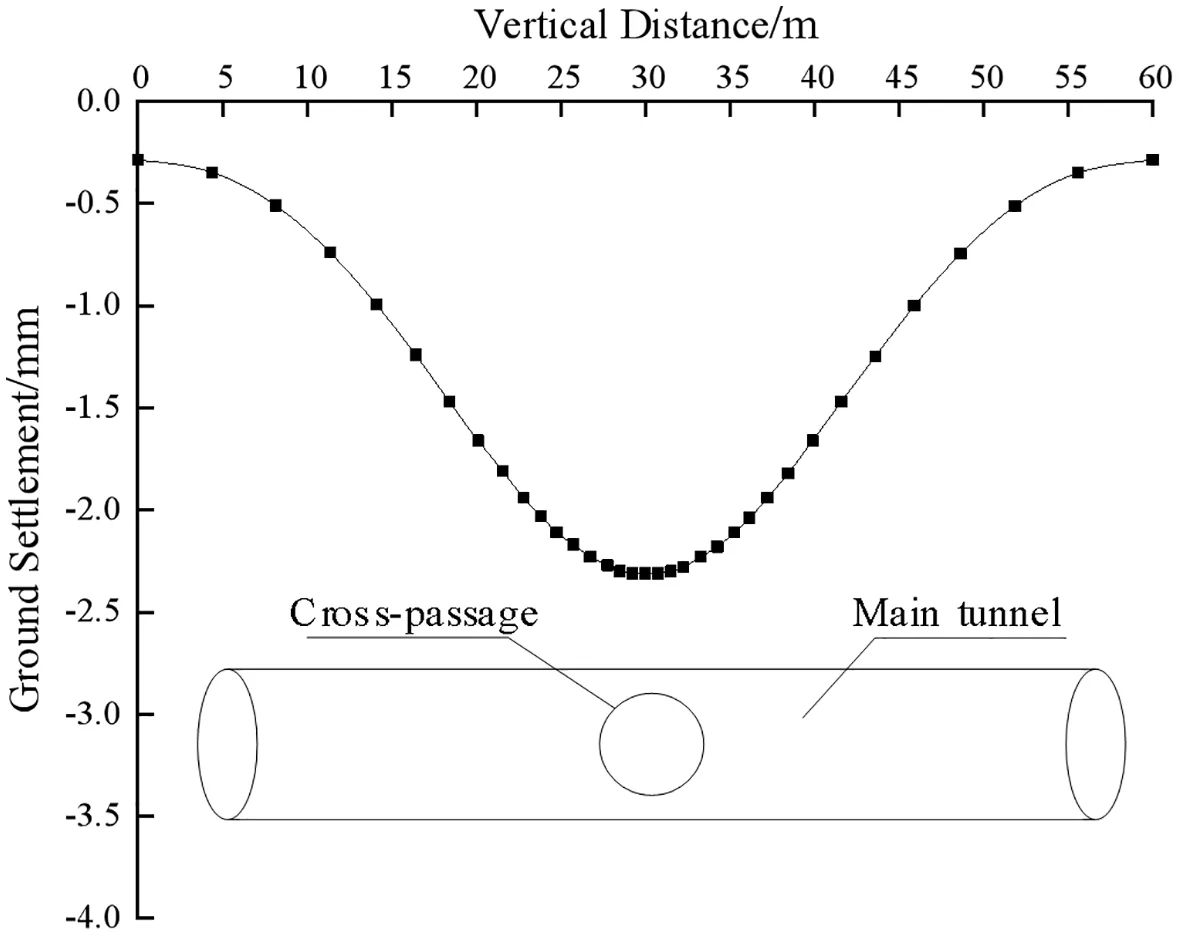
Longitudinal direction of the center line of the main tunnel.

**Fig 6:**
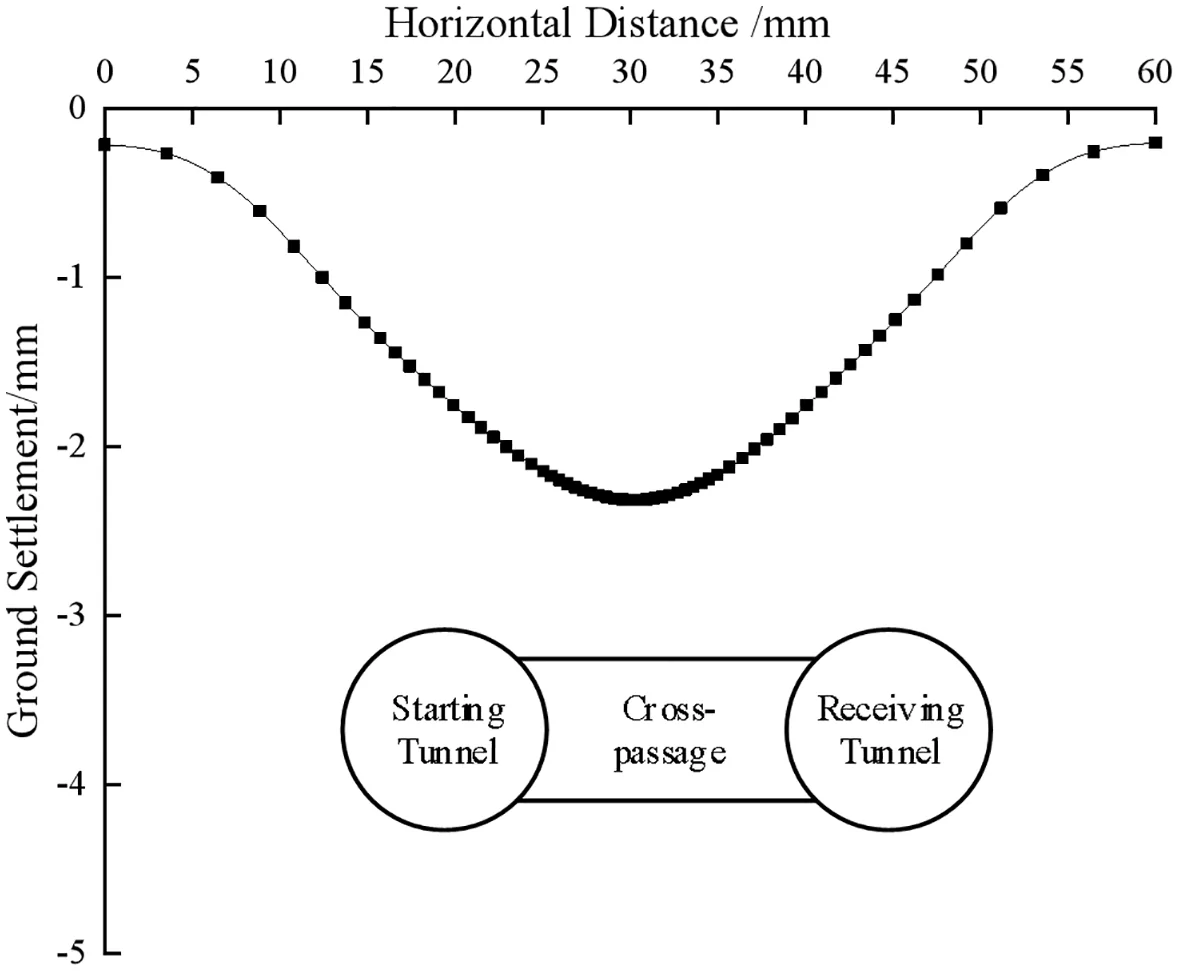
Longitudinal direction of the connecting passage.

Based on the above analysis, after the completion of the project, the curve presents an “inverted bell-shaped” characteristic, with the maximum settlement of 2.3 mm occurring at the center of the passage. The maximum longitudinal ground surface settlement along the main tunnel at the launching end is 1.8 mm, and the maximum longitudinal ground surface settlement along the main tunnel at the receiving end is 1.8 mm.

### 3.2 Structural deformation of left and right main line tunnels

To save space, only the deformation nephograms after construction are listed, as shown in Figs 7–12 respectively.

**Fig 7:**
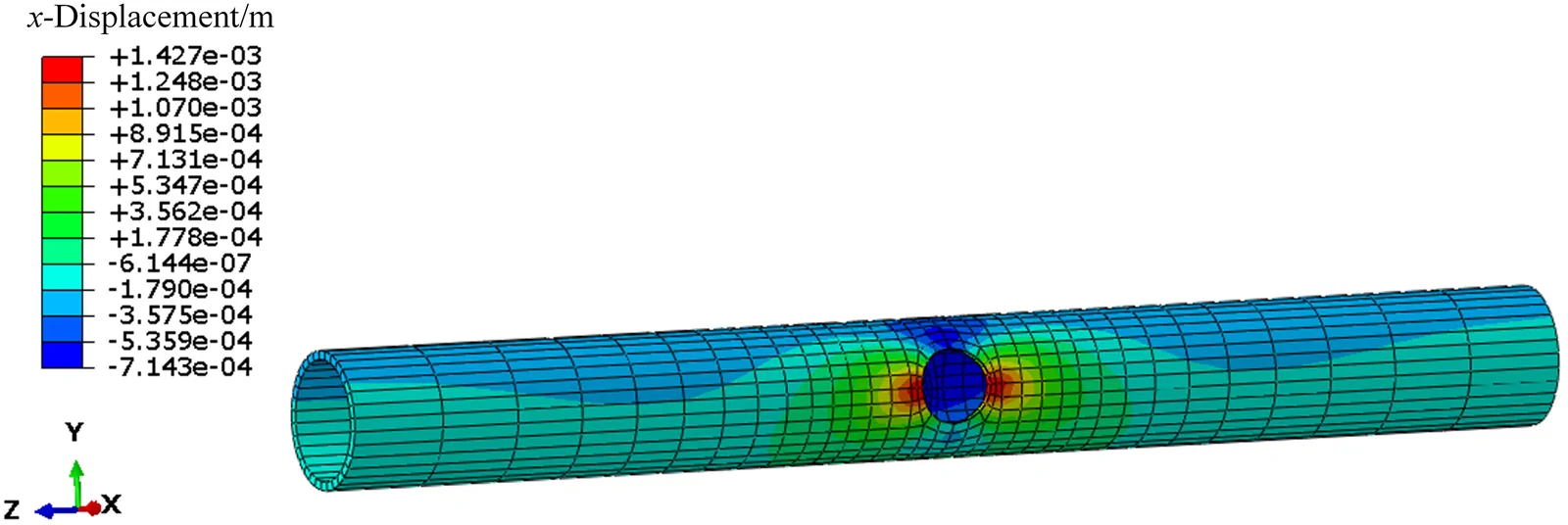
X-direction displacement.

**Fig 8:**
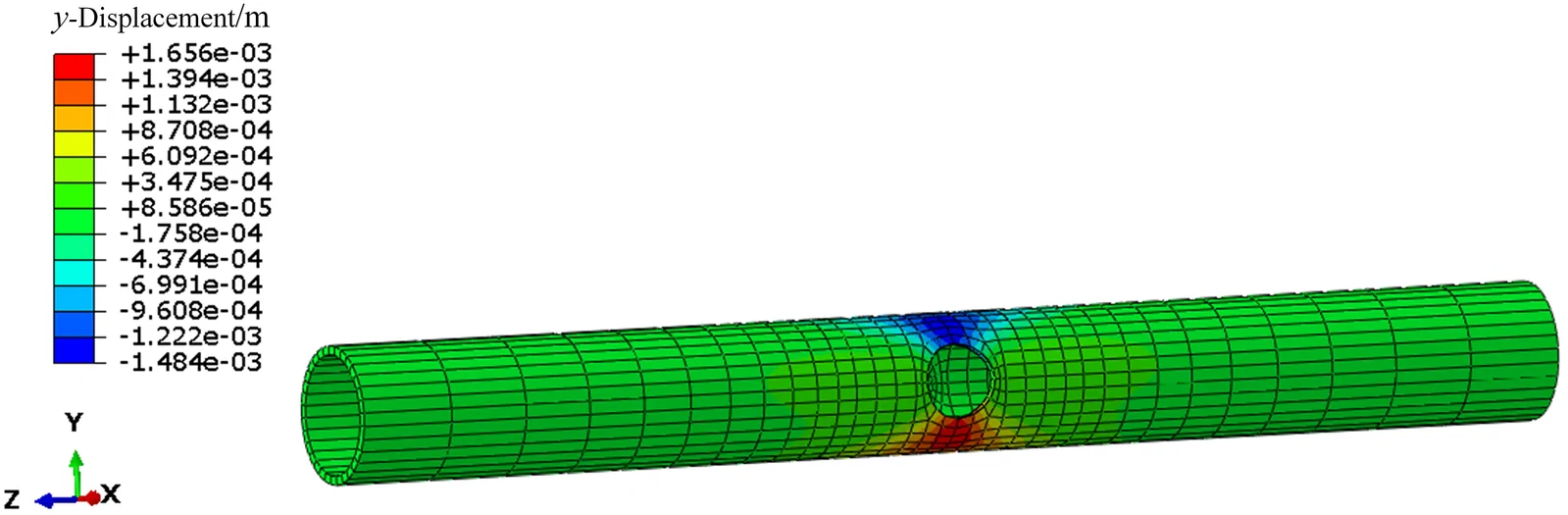
Y-direction displacement.

**Fig 9:**
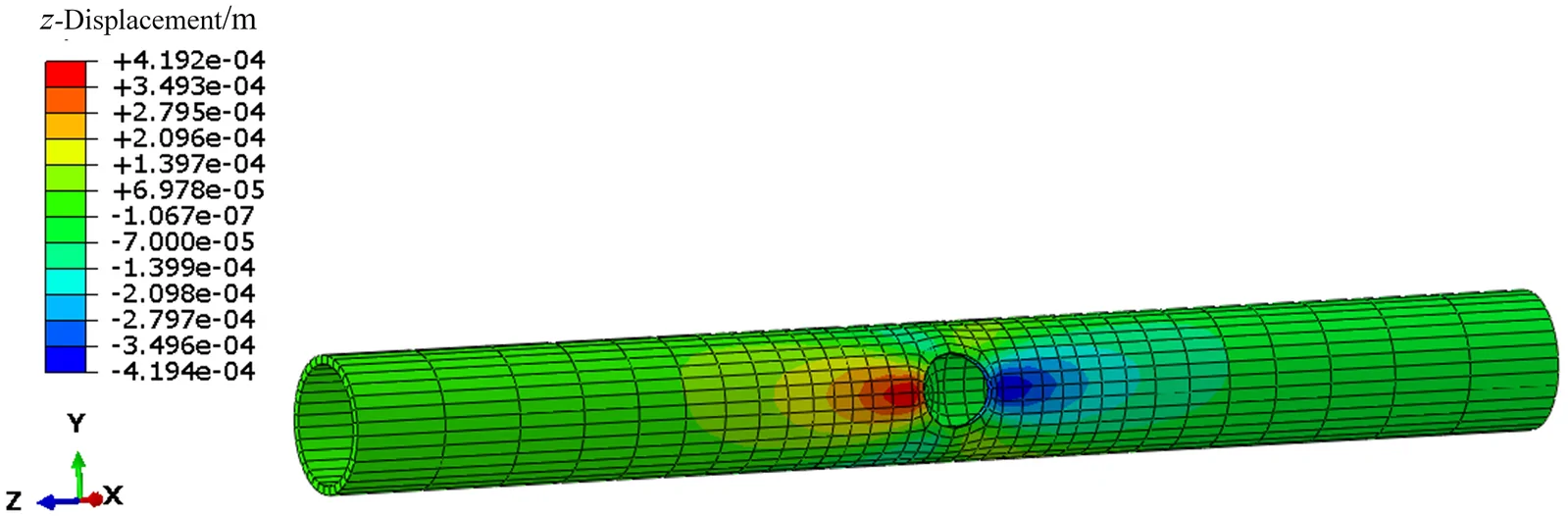
Z-direction displacement.

**Fig 10:**
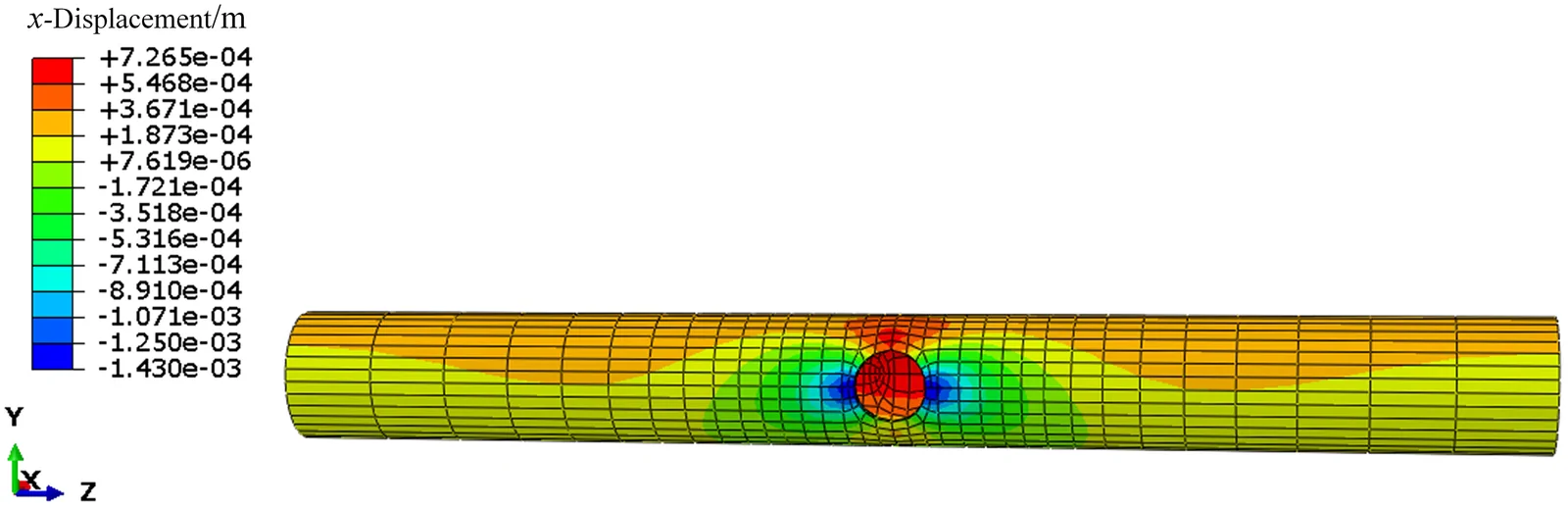
X-direction displacement.

**Fig 11:**
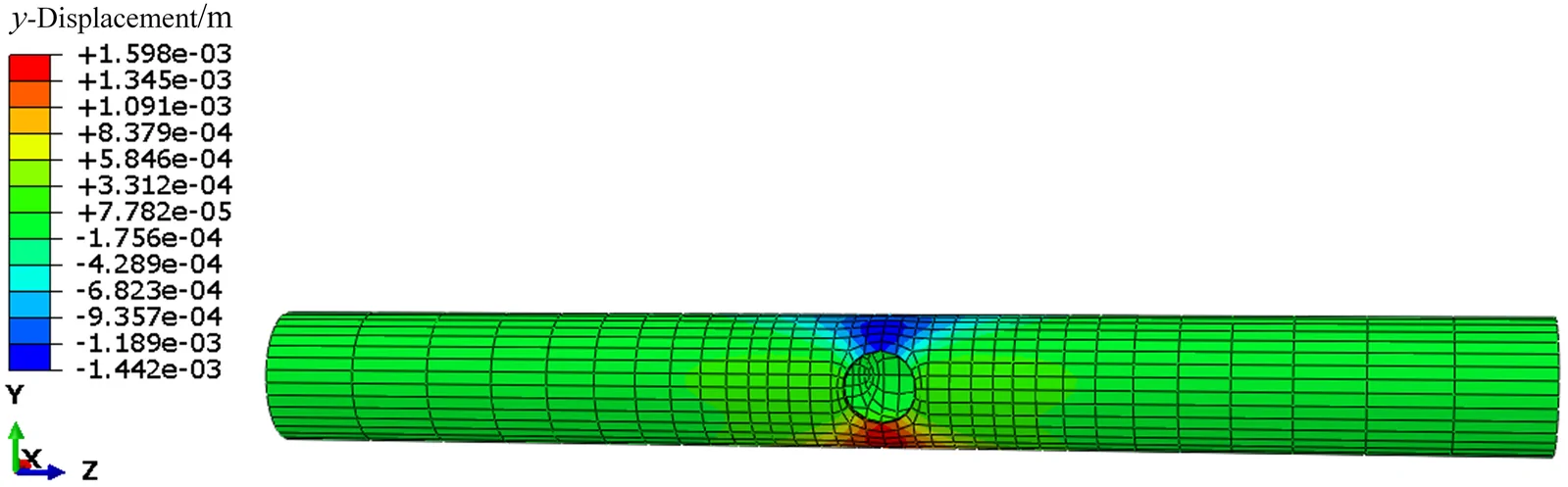
Y-direction displacement.

**Fig 12:**
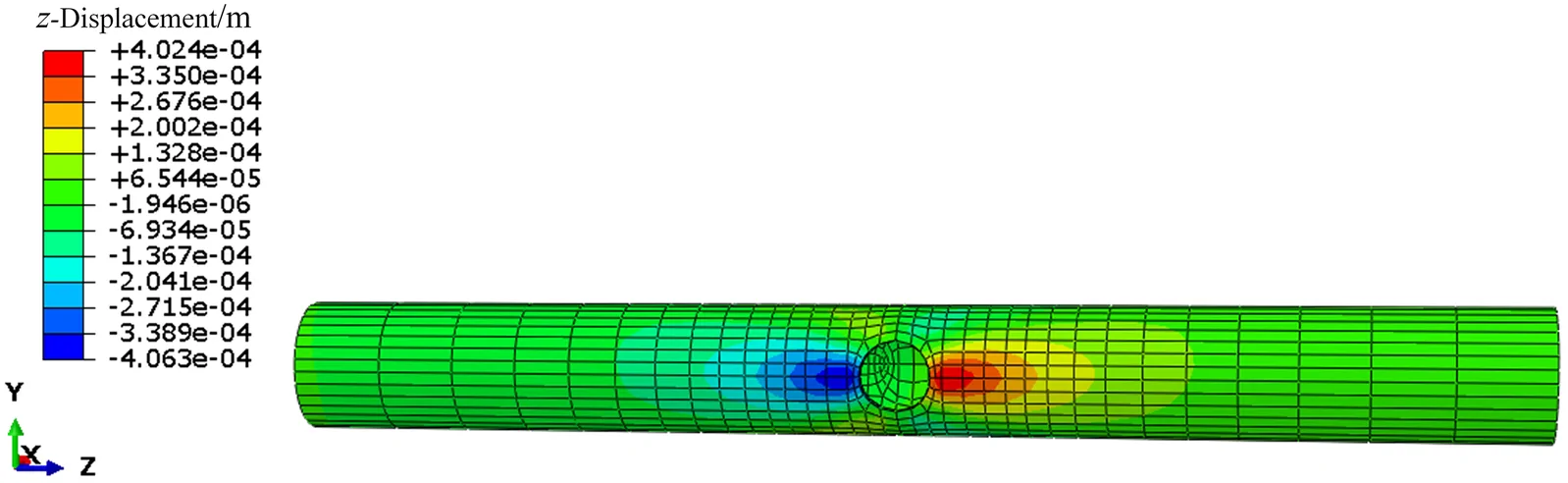
Z-direction displacement.

It can be seen from Figs 7-9 and Figs 10-12 that the deformation of the main tunnel structure caused by the mechanized construction of the connecting passage is dominated by vertical deformation and horizontal deformation along the longitudinal direction of the connecting passage. After construction, the maximum vertical deformation of the main tunnel structure at the launching end is 1.7 mm, the maximum horizontal deformation is 1.4 mm, and the maximum longitudinal deformation is 0.4 mm; the maximum vertical deformation of the main tunnel at the receiving end is 1.6 mm, the maximum horizontal deformation is 1.4 mm, and the maximum longitudinal deformation is 0.4 mm. The maximum horizontal deformation of the main tunnel occurs at the arch waist of the opening, which is caused by the unloading of the lateral soil at the opening and the reduction of constraints, resulting in the maximum horizontal deformation at the arch waist of the opening of the main tunnel.

### 3.3 Calculation results of tunnel structure internal force

According to the numerical calculation results, the bending moment and axial force values of the cutting ring at each construction stage were extracted, and the internal force distribution diagrams were drawn. To save space, only the internal force distribution after construction is listed.

#### 3.3.1 Bending moment.

The bending moment of the main line tunnel is shown in Figs 13–16, and the maximum value curve is shown in Fig 17.

**Fig 13:**
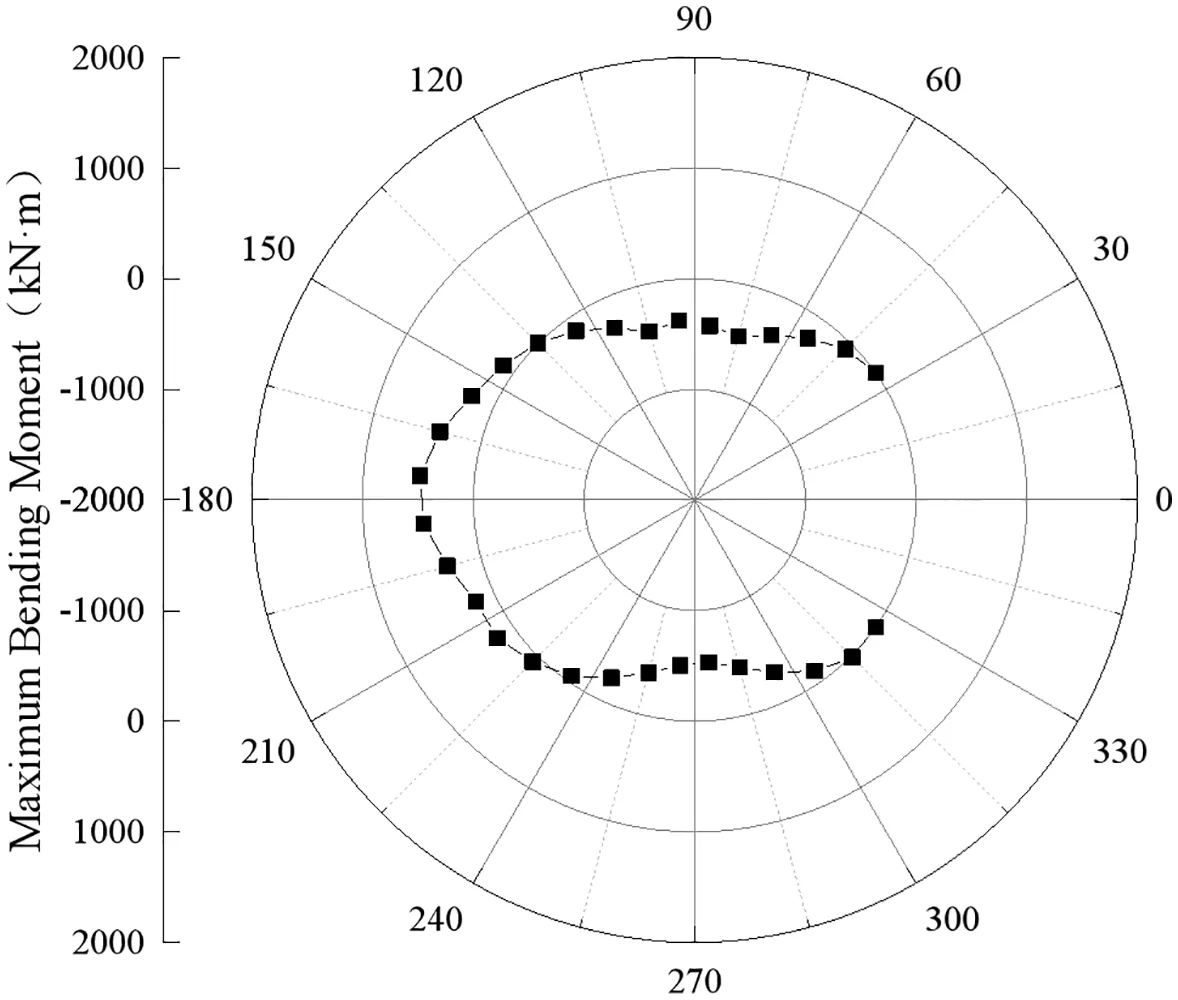
Full cutting ring (launching end).

**Fig 14:**
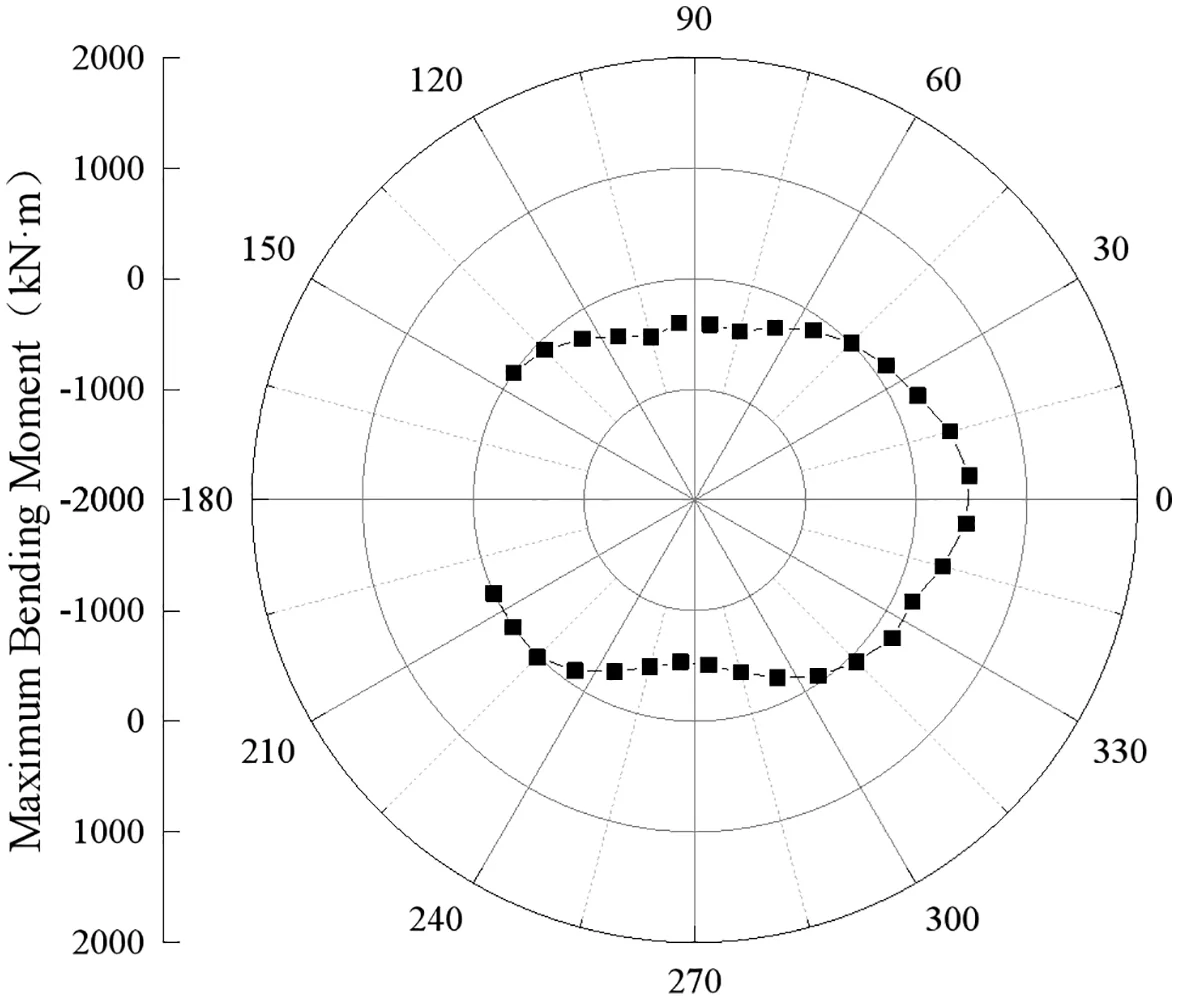
Full cutting ring (receiving end).

**Fig 15:**
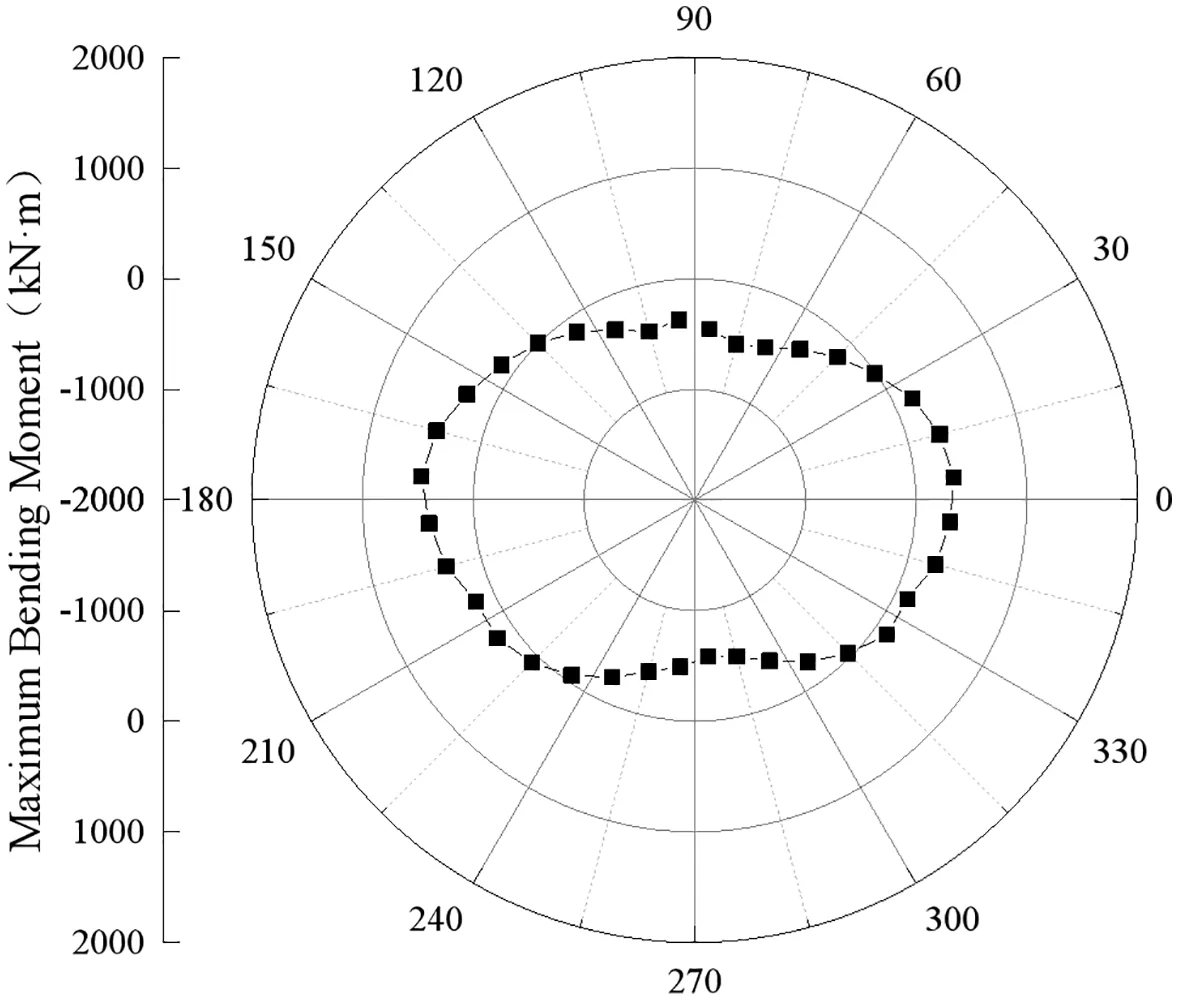
Half cutting ring (launching end).

**Fig 16:**
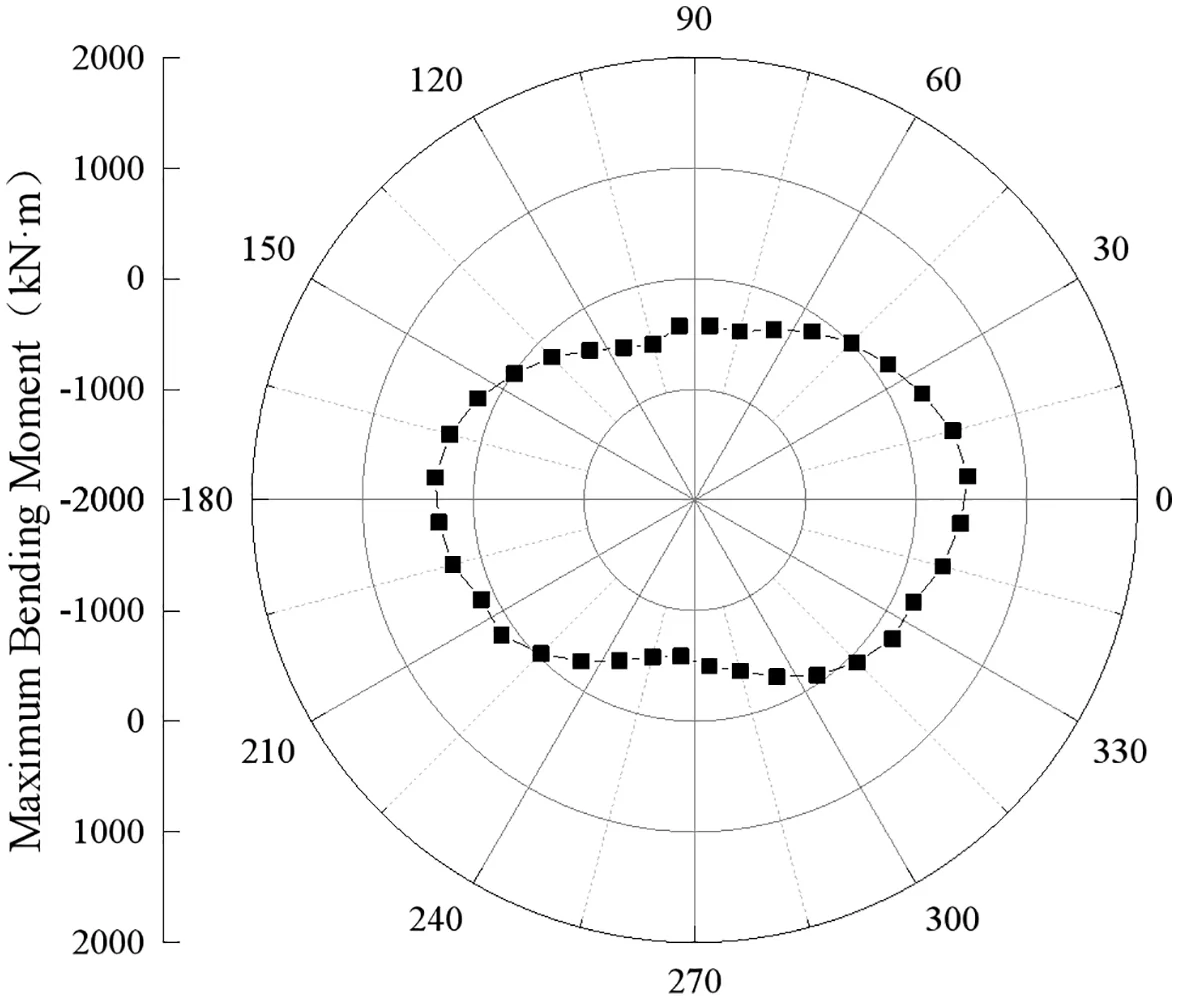
Half cutting ring (receiving end).

**Fig 17:**
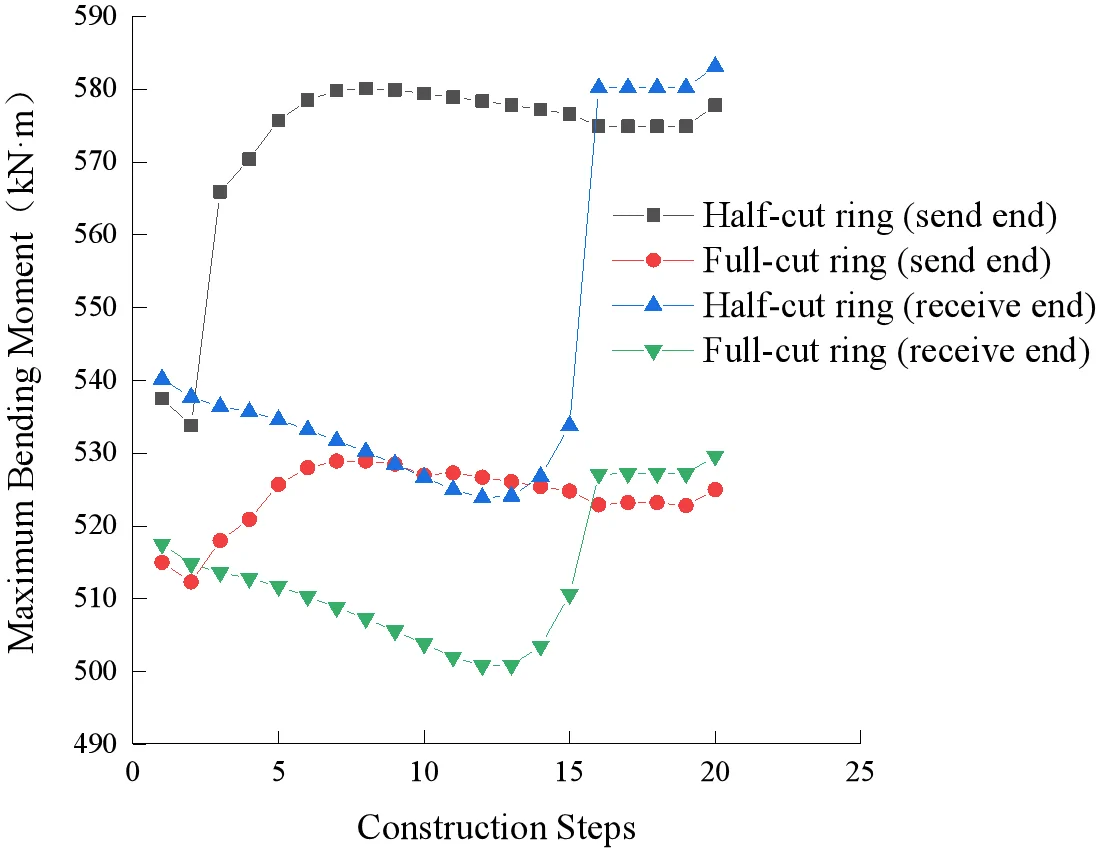
Maximum value curve (Unit: kN·m).

Based on the above analysis:

Before pipe jacking launching, during the internal support loading stage, the maximum bending moment of the cutting ring decreased by 2.5 ~ 4 kN·m; when the launching portal was broken, the maximum bending moment of the half cutting ring at the launching end increased sharply by 32.1 kN·m; from the launching and advancing of the pipe jacking to before the breaking of the receiving portal, the maximum bending moment of the cutting ring showed a trend of first increasing then decreasing and first decreasing then increasing, respectively; when the receiving portal was broken, the maximum bending moment of the cutting ring at the receiving end increased sharply by 46.4 kN·m; during the internal support unloading stage, the maximum bending moment of the cutting ring increased by 2 ~ 3 kN·m.

#### 3.3.2 Axial force.

The axial force of the main line tunnel is shown in Figs 18–21, and the maximum value curve is shown in Fig 22.

**Fig 18:**
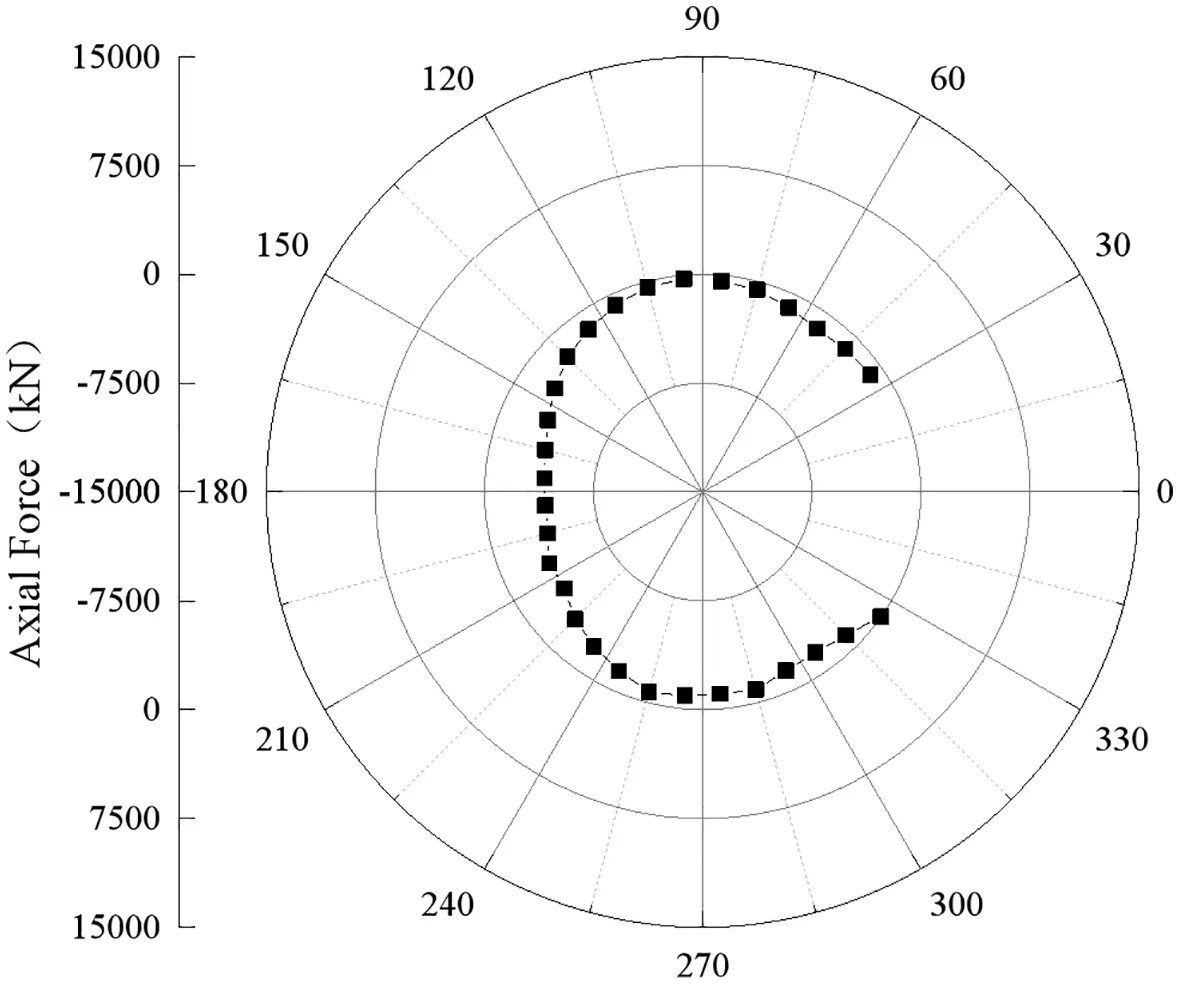
Full cutting ring (launching end).

**Fig 19:**
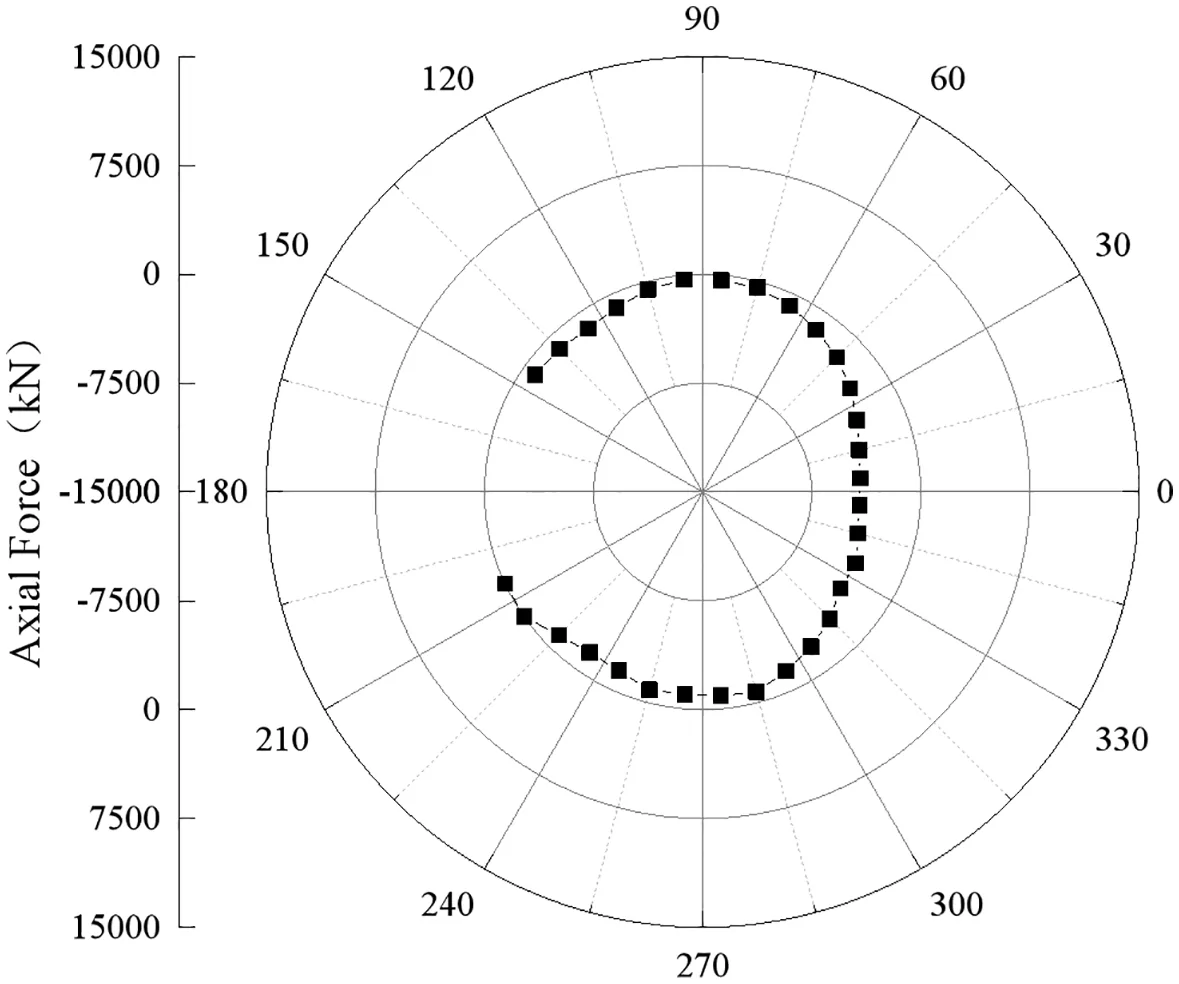
Full cutting ring (receiving end).

**Fig 20:**
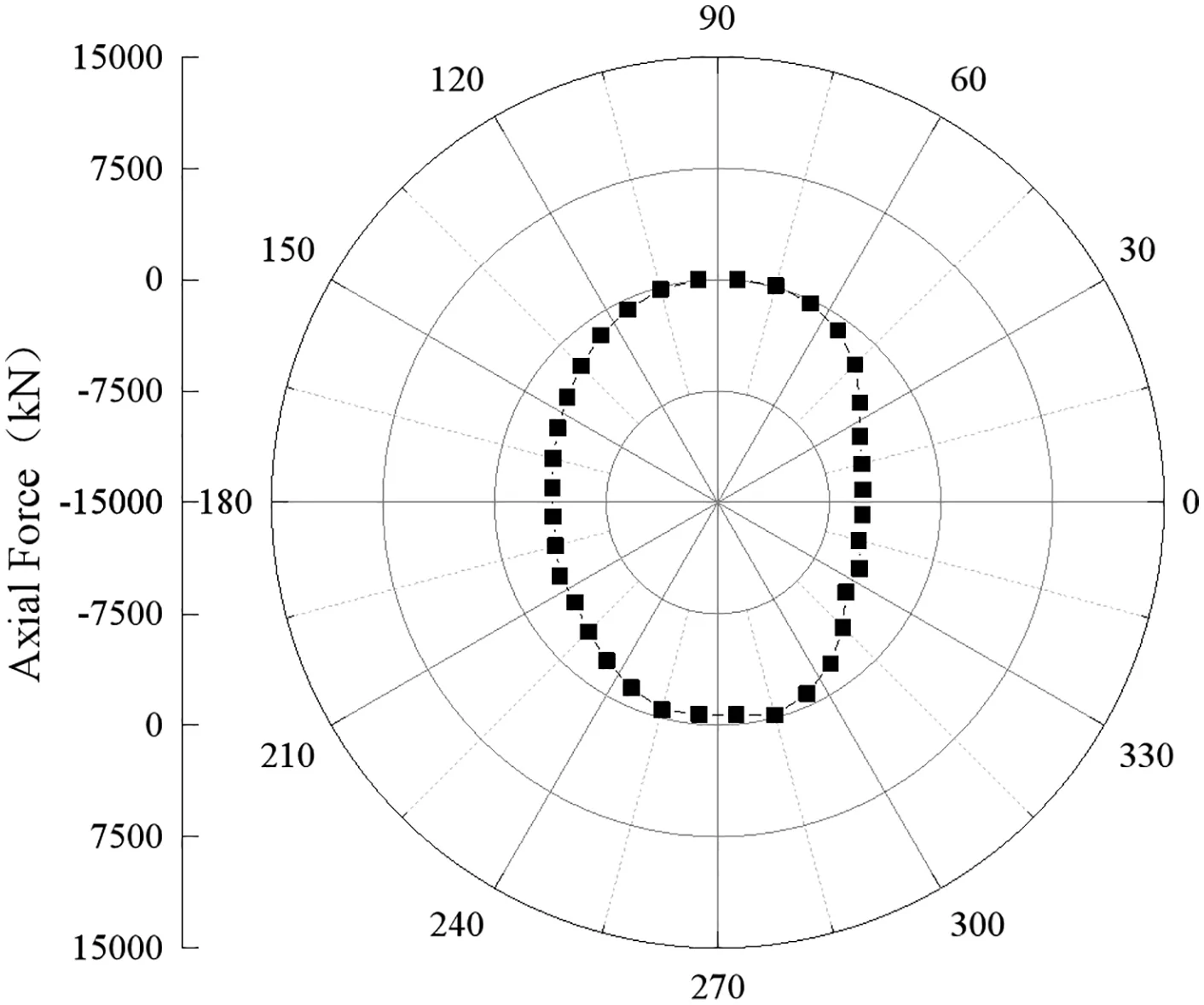
Half cutting ring (launching end).

**Fig 21:**
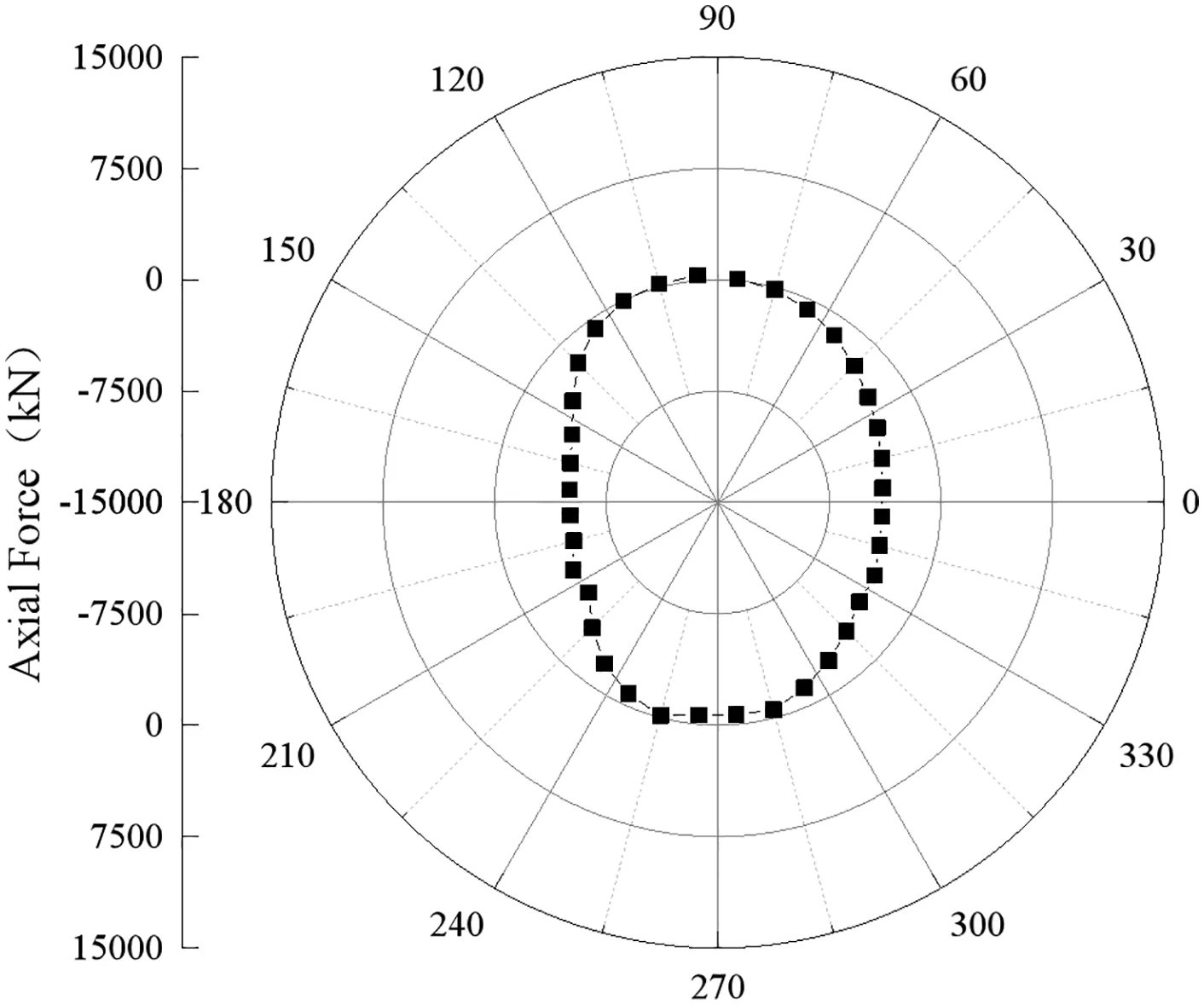
Half cutting ring (receiving end).

**Fig 22:**
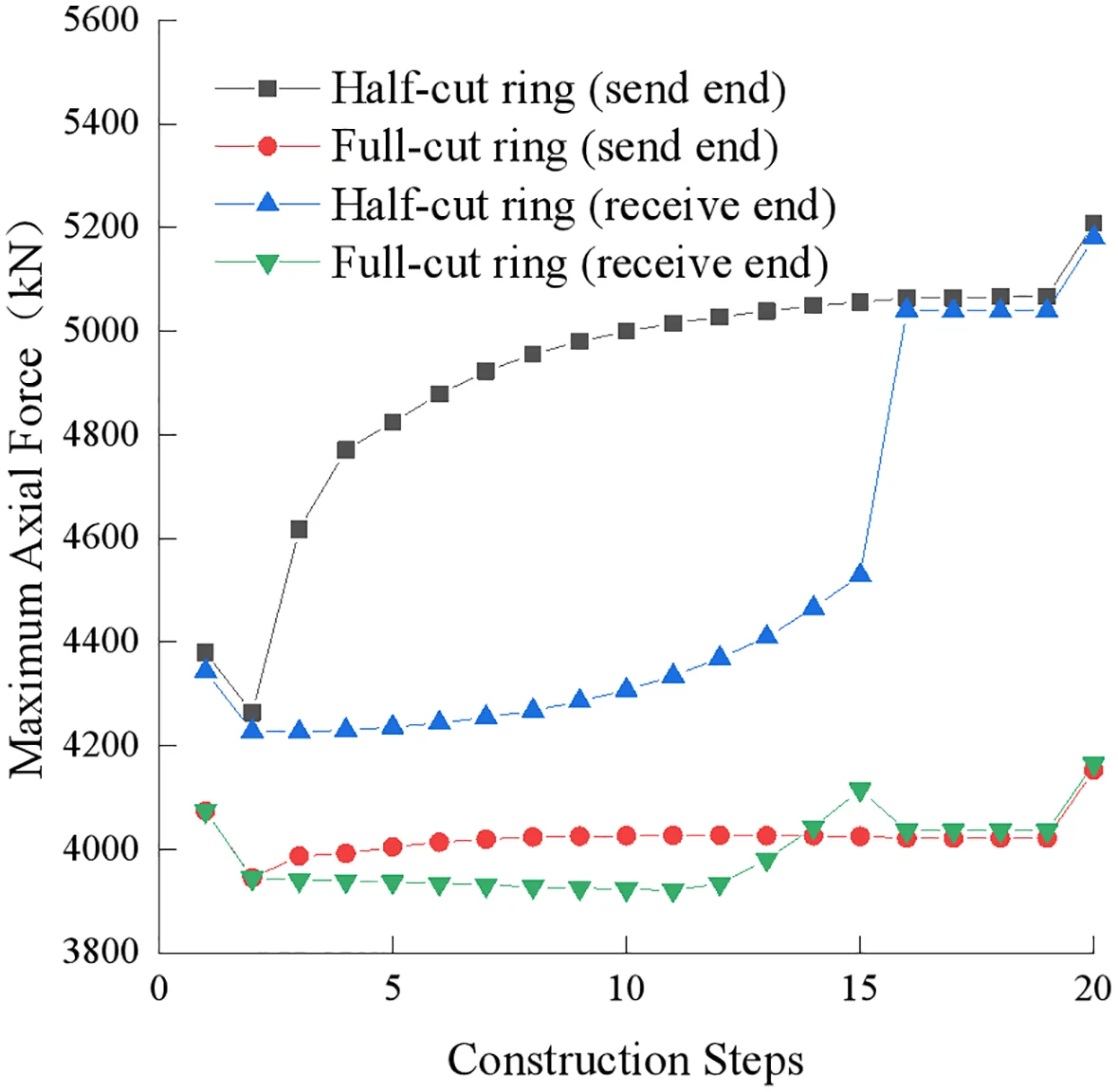
Maximum value curve (Unit: kN).

Based on the above analysis:

Before pipe jacking launching, during the internal support loading stage, the maximum axial force of the cutting ring decreased by 115 ~ 130 kN; when the launching portal was broken, the maximum axial force of the half cutting ring at the launching end increased sharply by 354 kN; from the launching and advancing of the pipe jacking to before the breaking of the receiving portal, the axial force of the cutting ring basically showed an increasing trend, but the growth rate at the launching end gradually slowed down, while that at the receiving end gradually increased; when the receiving portal was broken, the maximum axial force of the cutting ring at the receiving end increased sharply by 512 kN; during the internal support unloading stage, the maximum axial force of the cutting ring at the launching and receiving ends increased by 125 ~ 145 kN.

## 4. Result comparison and verification

### 4.1 Verification of ground surface settlement results

During the construction of the connecting passage, the ground surface settlement presented an “inverted bell-shaped” characteristic. Referring to the Peck formula [14], its logarithmic form is shown in Equation (1):


$$\begin{array}{c} lnS(x)=lnS_{max}+(1/k^{2})(-x^{2}/2) \end{array}$$ (1)

Through regression analysis and substitution of monitoring data, the Peck curve equation in logarithmic form along the longitudinal direction of the main tunnel center line is obtained:


$$\begin{array}{c} lnS(x)=0.81+0.015(-x_{i}^{2}/2) \end{array}$$ (2)

Where: *S(x)* is the ground surface settlement at x from the tunnel center, mm; *S*_*max*_ is the maximum settlement at the tunnel center, mm; *k* is the width coefficient of the settlement trough, m; *x* is the horizontal distance from the tunnel center, m; *x*_*i*_ is the distance between the i-th monitoring point and the tunnel axis, m.

The comparison curve between the fitted Peck curve and the numerical calculation results is shown in Fig 23 below.

**Fig 23:**
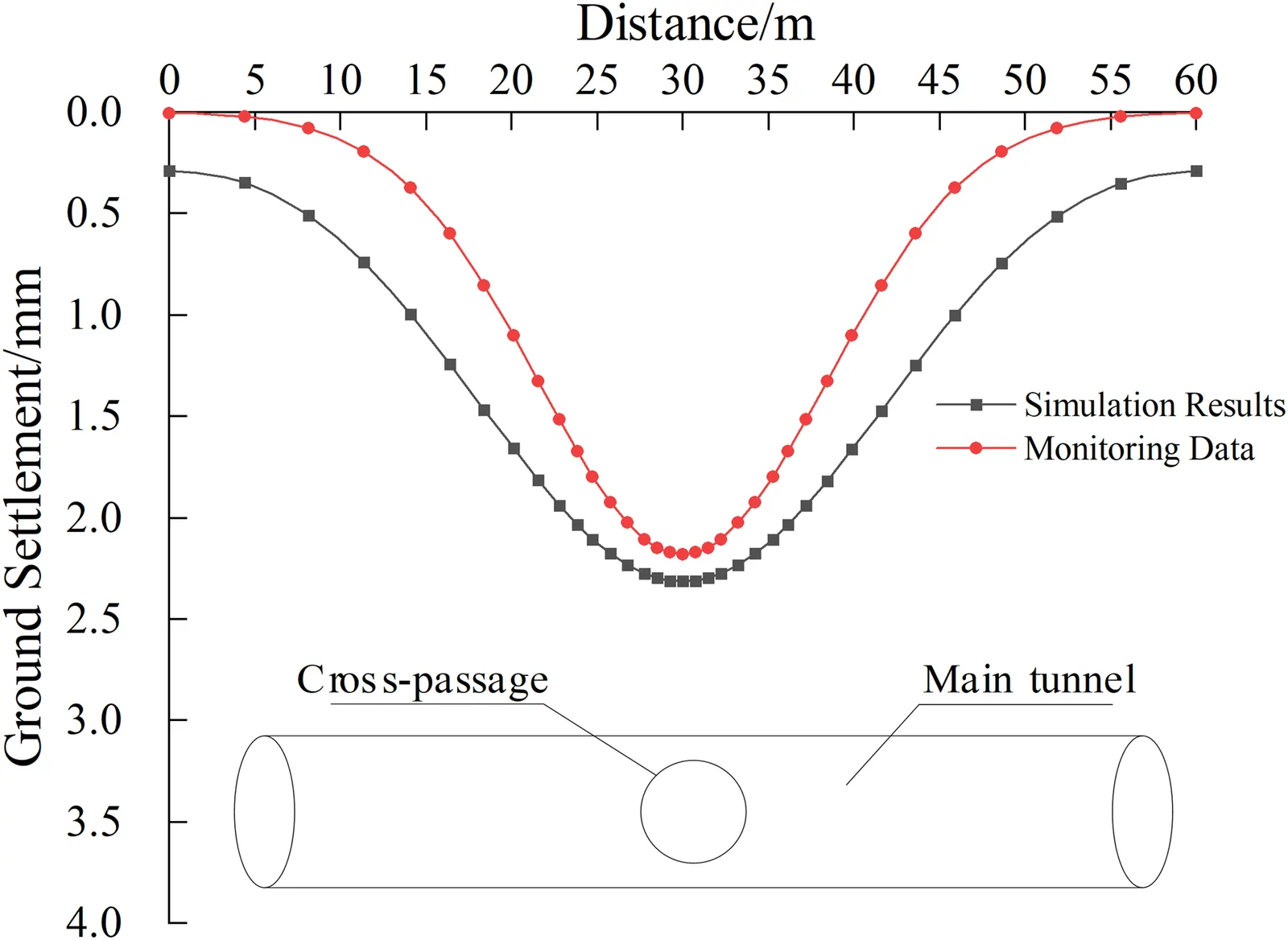
Comparison curve between simulation results and monitoring data.

Both the monitoring results and the calculation results show that the maximum ground settlement occurs directly above the center of the connecting passage.

The calculation results show that the maximum ground settlement is 2.3 mm, and the monitoring results show that the maximum ground settlement is 2.2 mm. It can be seen from the data trend comparison diagram that the two have a high fitting degree and consistent variation trend, which proves the accuracy of the numerical analysis software.

### 4.2 Internal force monitoring results

During the construction, the stress of the tunnel structure was monitored, and the schematic diagram of the monitoring point layout is shown in Figs 24 and 25. The comparison results of axial force and bending moment between monitoring data and numerical simulation are shown in Figs 26–29.

**Fig 24:**
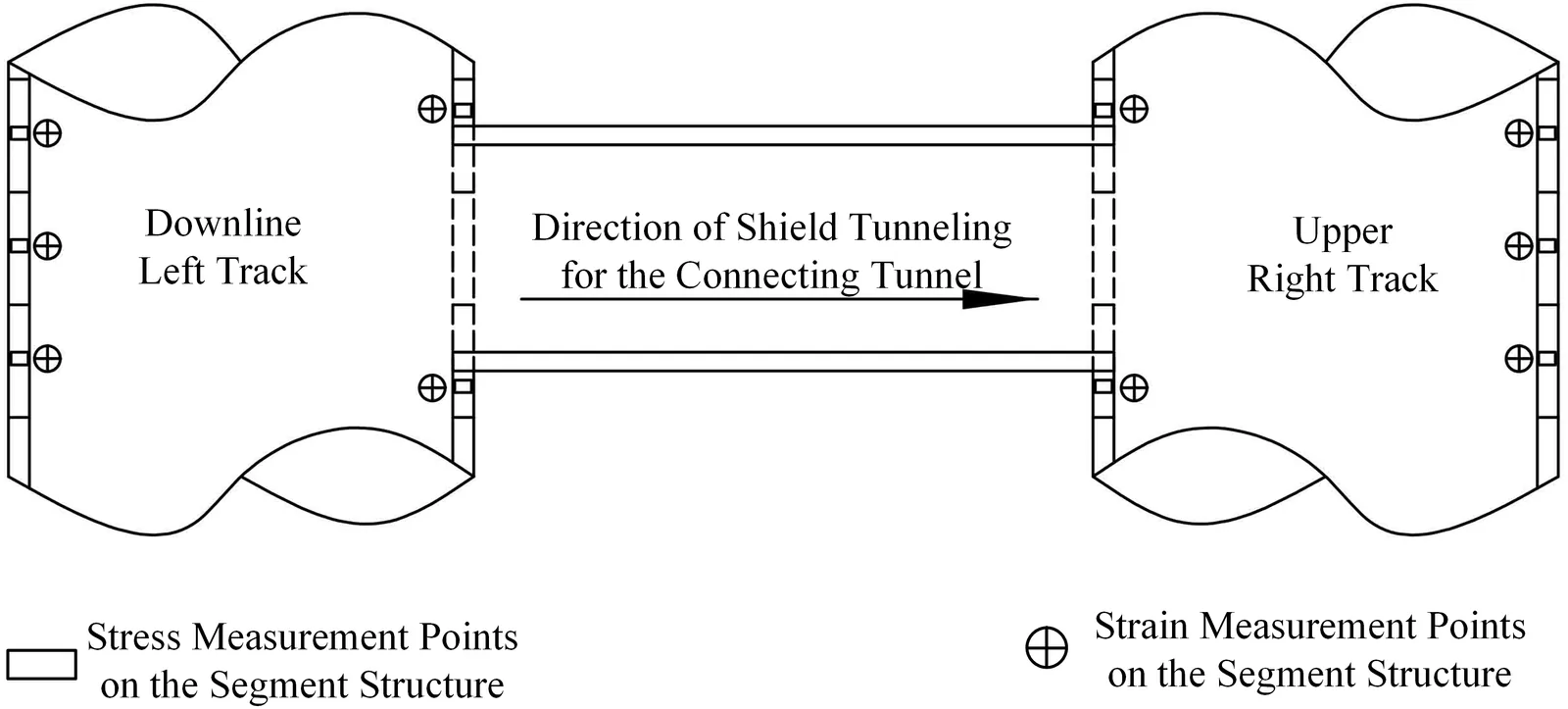
Plan schematic diagram.

**Fig 25:**
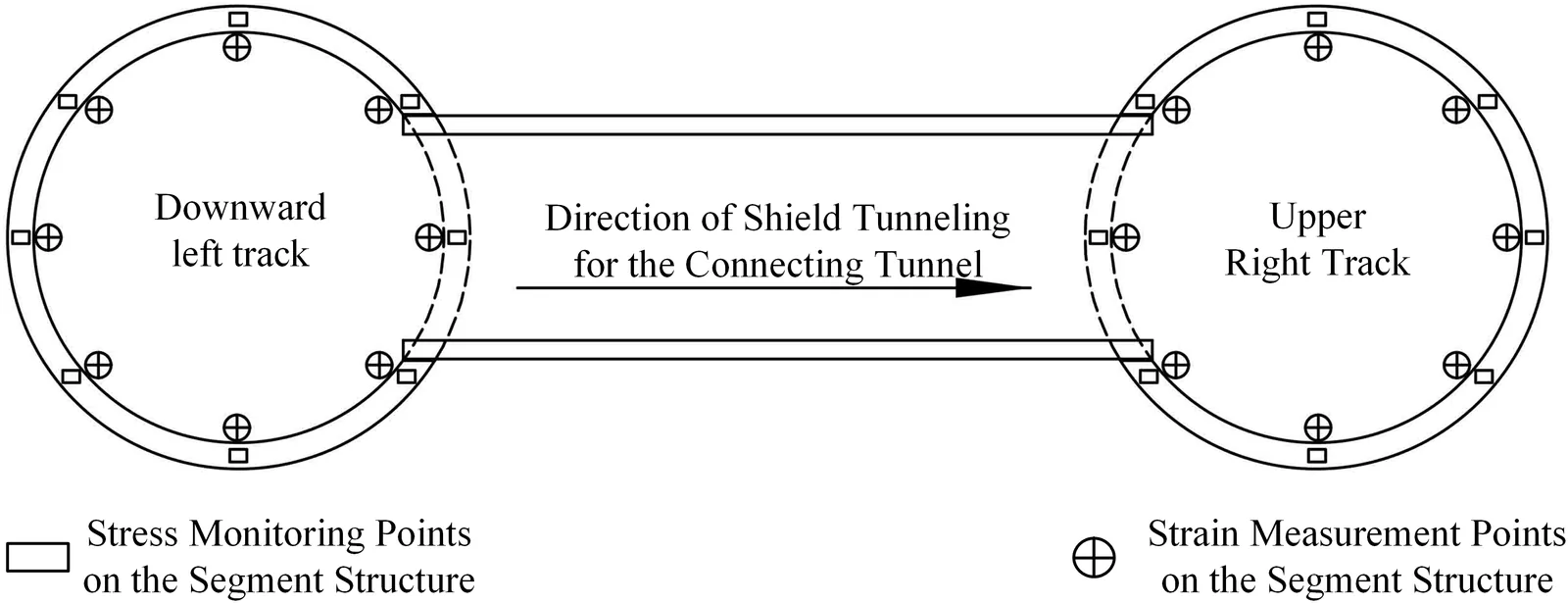
Profile schematic diagram.

**Fig 26:**
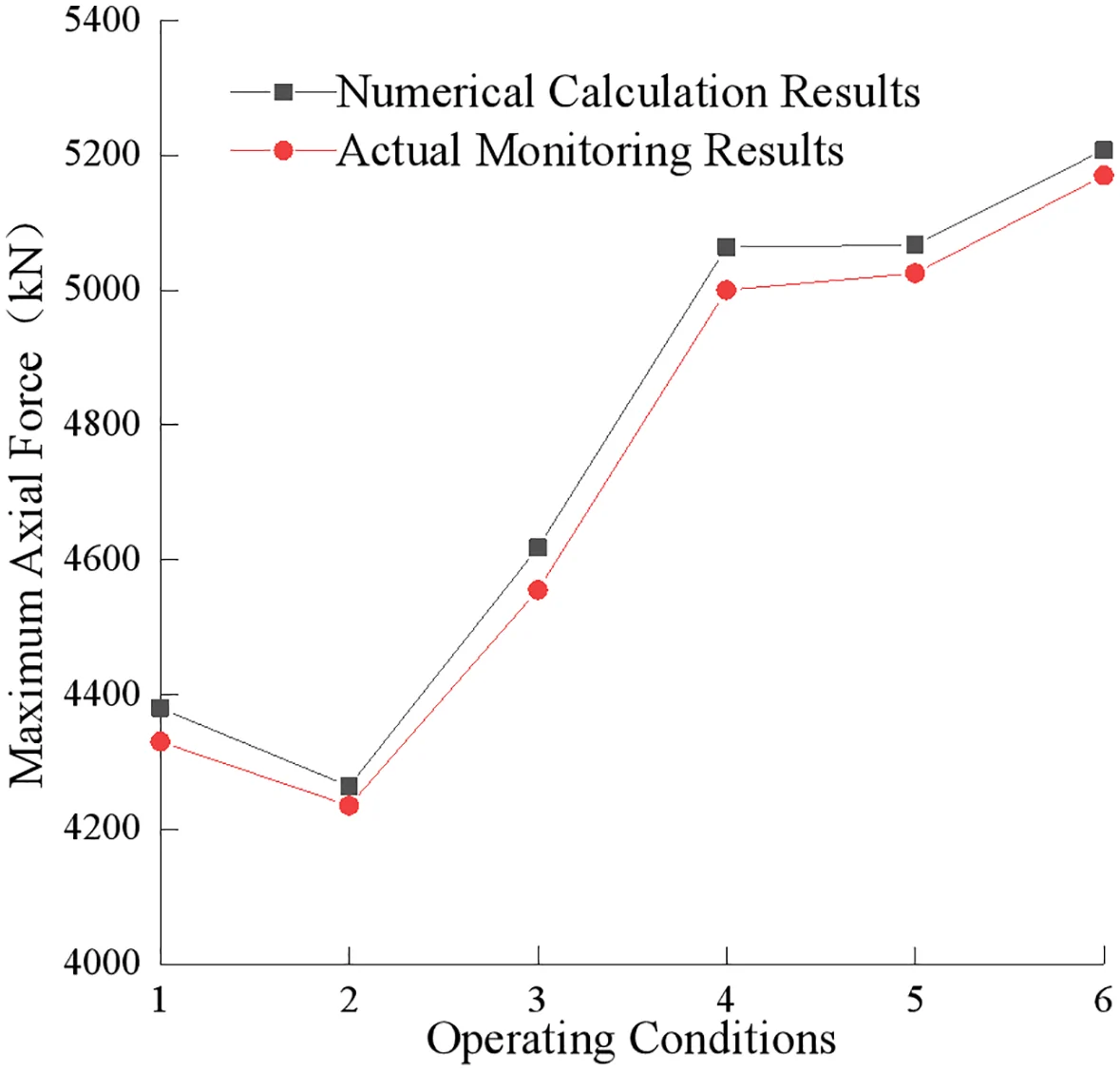
Axial force (launching end).

**Fig 27:**
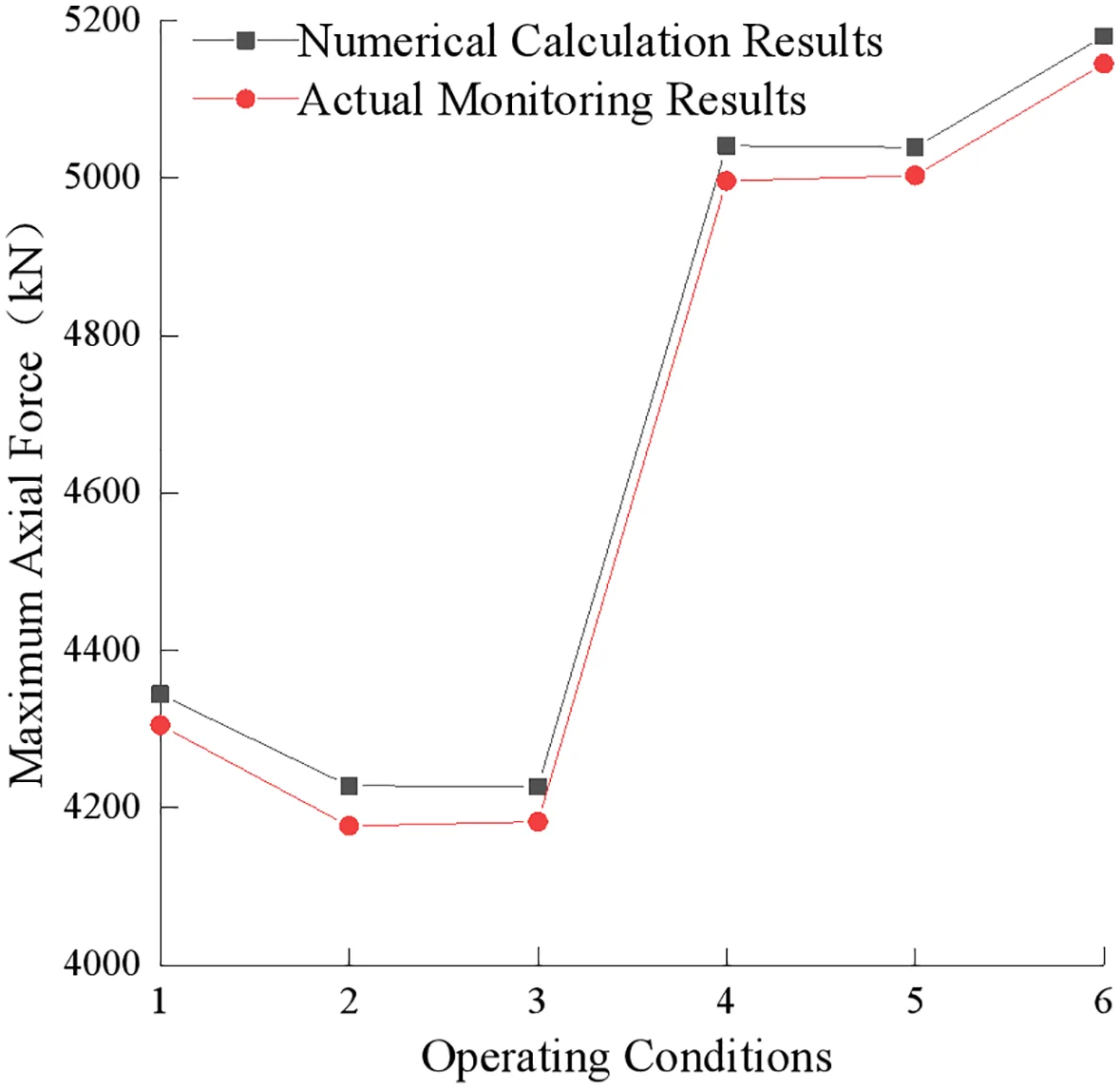
Axial force (receiving end).

**Fig 28:**
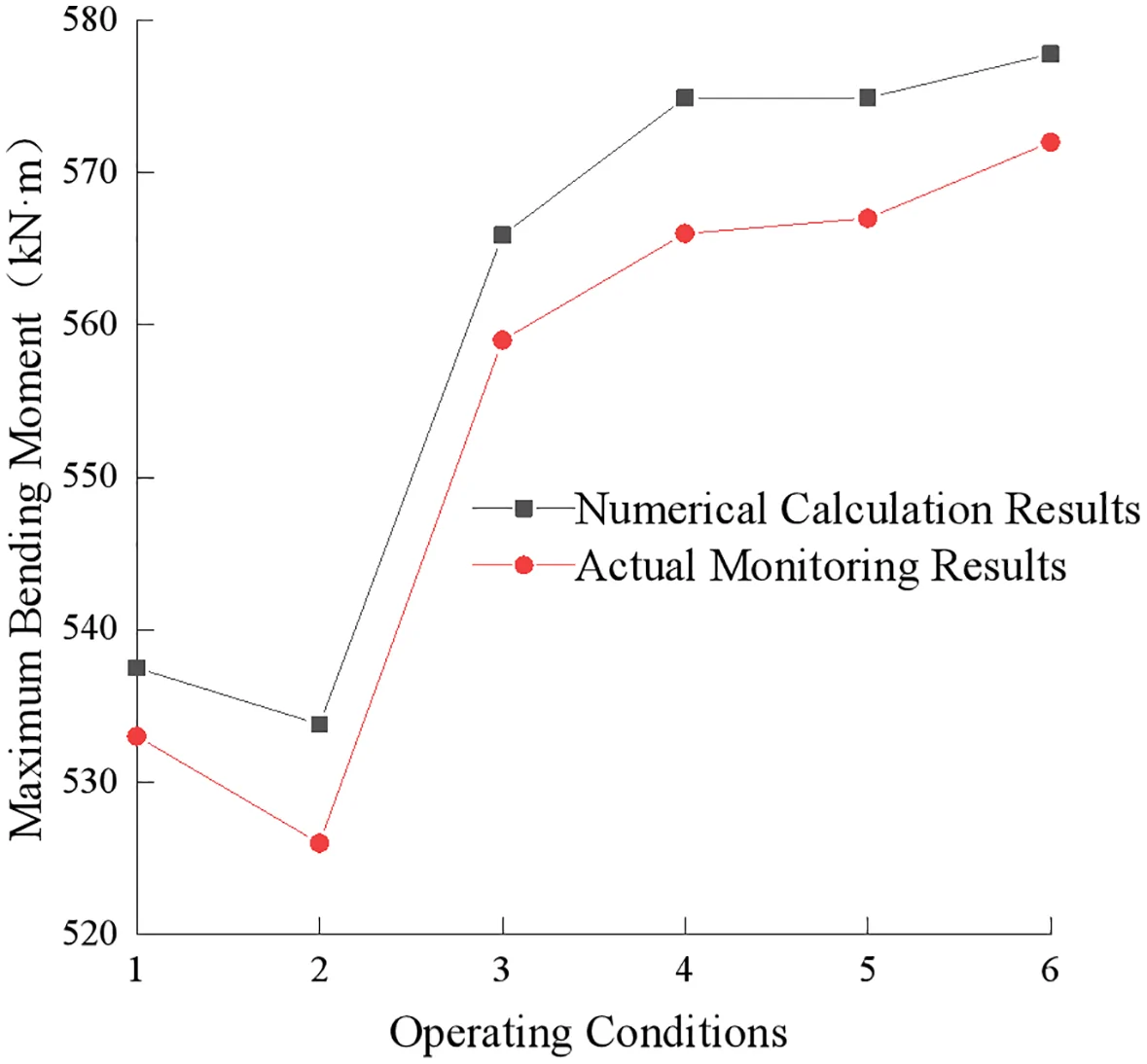
Bending moment (launching end).

**Fig 29:**
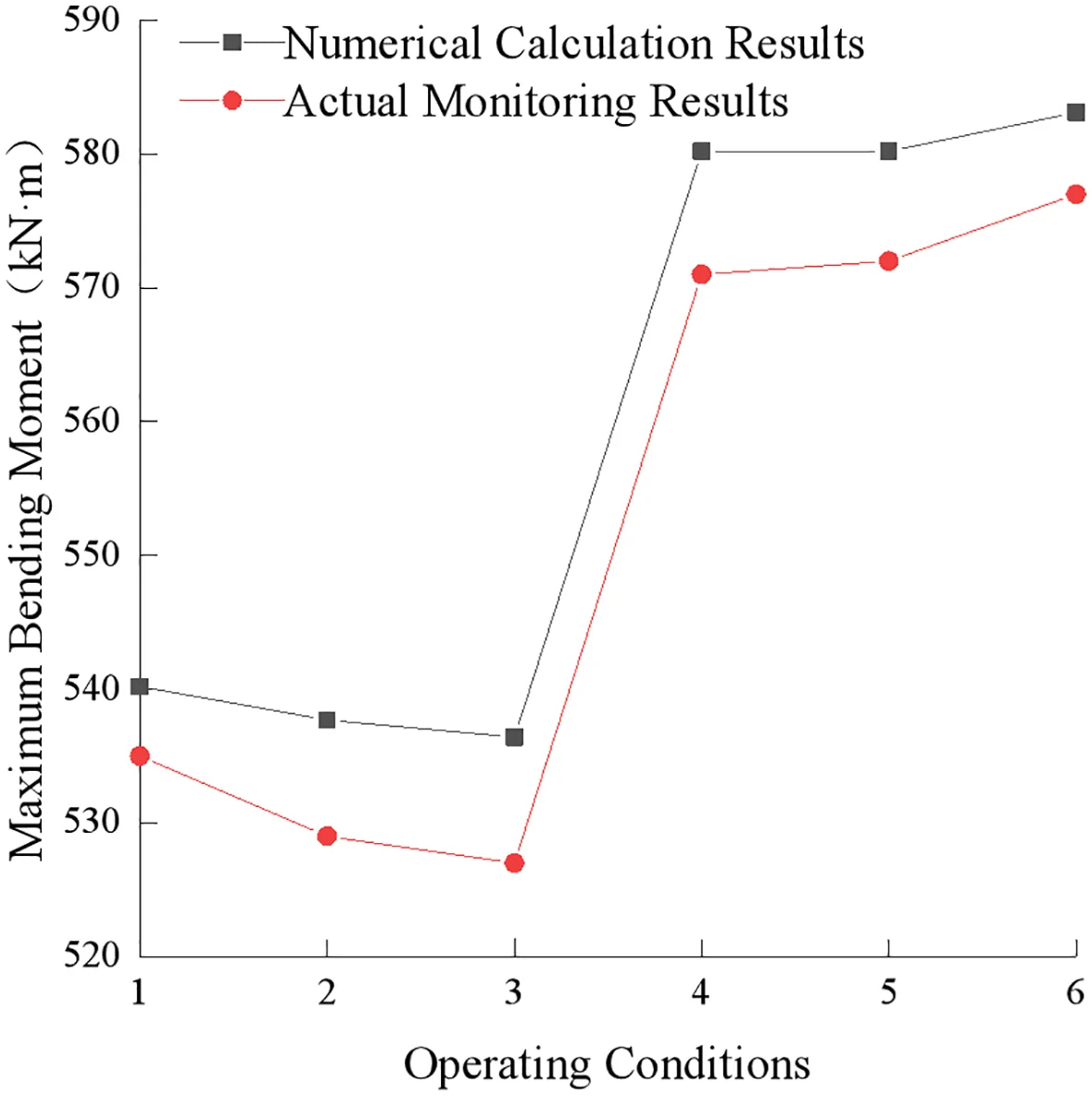
Bending moment (receiving end).

Based on the above analysis:

(1) Launching end

From Working Condition 1 to Working Condition 2, considering the support effect, the maximum bending moment decreased by 7 kN·m, and the maximum axial force decreased by 7 kN·m and 95 kN respectively;

From Working Condition 2 to Working Condition 4, the maximum bending moment increased by 40 kN·m, and the maximum axial force increased by 765 kN;

From Working Condition 5 to Working Condition 6, considering the support removal, the maximum bending moment increased by 5 kN·m, and the maximum axial force increased by 145 kN.

(2) Receiving end

From Working Condition 1 to Working Condition 2, considering the support effect, the maximum bending moment decreased by 6 kN·m, and the maximum axial force decreased by 128 kN;

From Working Condition 2 to Working Condition 4, the maximum bending moment increased by 42 kN·m, and the maximum axial force increased by 819 kN;

From Working Condition 5 to Working Condition 6, considering the support removal, the maximum bending moment increased by 5 kN·m, and the maximum axial force increased by 142 kN.

It can be seen from the data trend comparison diagram that the two have a high fitting degree and consistent variation trend, which further proves the accuracy of the numerical analysis software.

## 5 Influence analysis of different construction parameters

To study the influence of different parameters on the mechanized construction of connecting passages, the single variable control method was adopted to systematically analyze the influence laws of mechanical parameters (cohesion, internal friction angle) of completely weathered granite stratum and spatial parameters (spacing between main line tunnels, burial depth) on the mechanized construction of connecting passages. By establishing numerical models and adjusting the above parameters in turn, the ground surface settlement in the projection area of the connecting passage axis and the maximum horizontal and vertical displacements of the main line tunnel structure were taken as evaluation indexes to study the influence of different parameter changes on ground surface settlement and tunnel deformation.

### 5.1 Construction influence of different cohesion

To study the influence law of soil cohesion on the mechanized construction of connecting passages, five groups of comparison working conditions were established by adjusting the cohesion parameter c of the completely weathered granite soil layer in the material model, with a gradient of 5 kPa within the range of 20 ~ 40 kPa, to investigate the influence of soil cohesion change on the mechanical response of connecting passage construction.

#### 5.1.1 Influence of different cohesion on ground settlement.

The influence of different cohesion of weathered granite stratum on ground settlement is shown in Fig 30.

**Fig 30:**
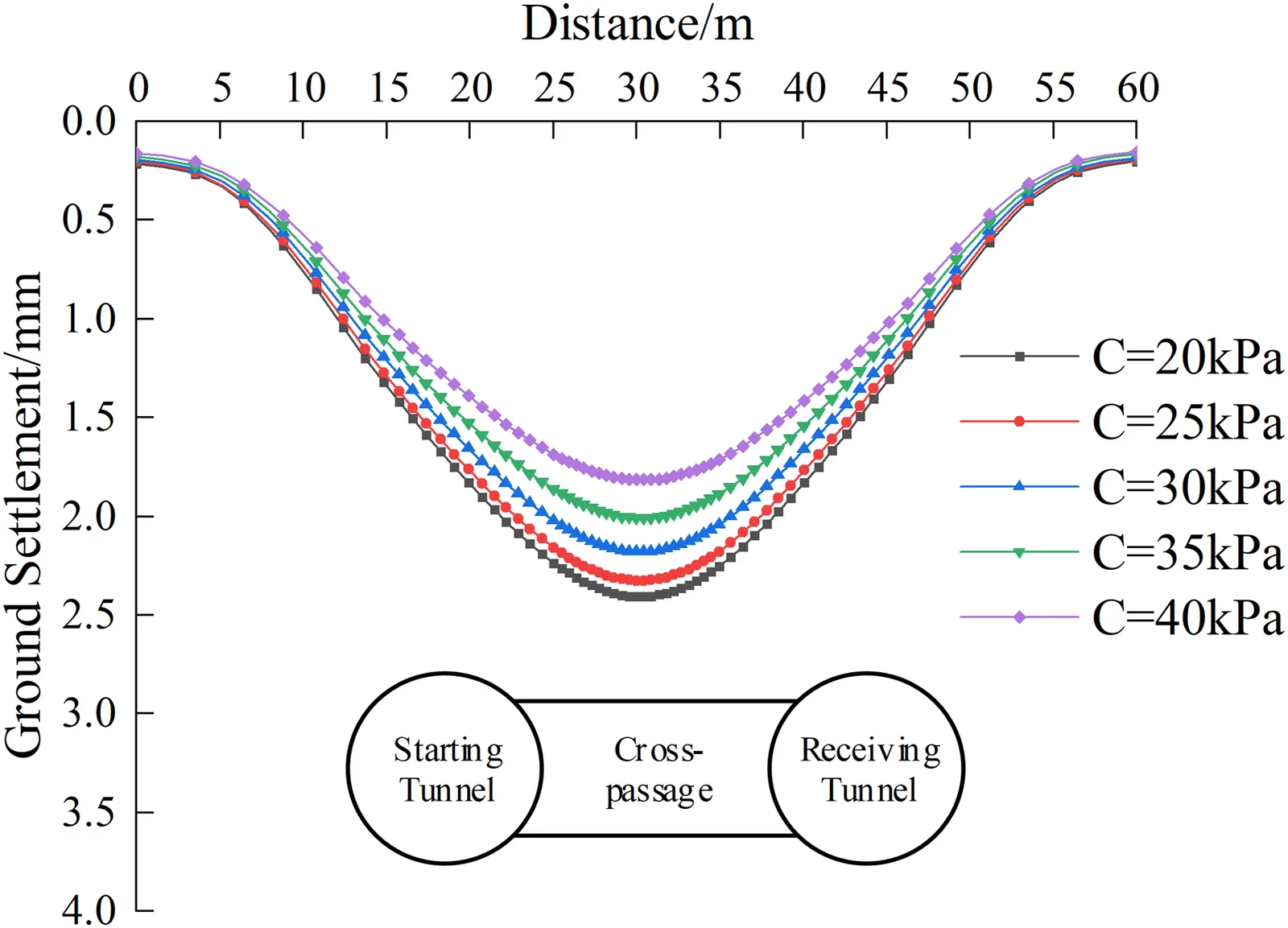
Ground settlement curves under different cohesion conditions.

It can be seen from Fig 30 that with the increase of the cohesion of the weathered granite stratum, the maximum ground settlement gradually decreases. When the cohesion of the weathered granite stratum increases from 20 kPa to 40 kPa, the maximum ground settlement decreases by 0.6 mm.

#### 5.1.2 Influence of different cohesion on deformation of main line tunnel.

The influence of different cohesion of weathered granite stratum on the vertical and horizontal deformation of the main line tunnel is shown in Fig 31.

**Fig 31:**
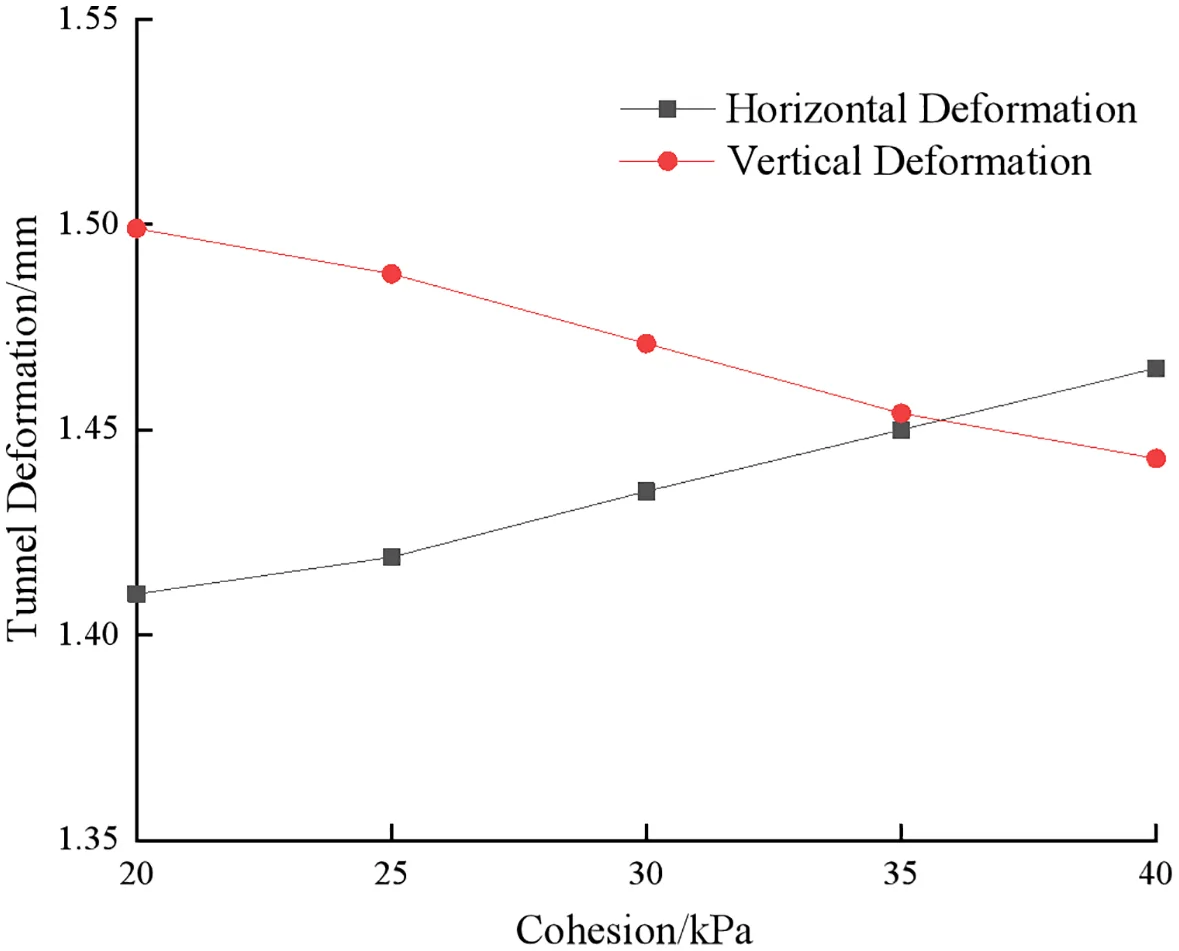
Deformation of the main line tunnel under different cohesion conditions.

It can be seen from Fig 31 that the maximum vertical deformation of the main line tunnel decreases linearly with the increase of the cohesion of the weathered granite stratum, while the maximum horizontal deformation of the main line tunnel increases linearly with the increase of the cohesion of the weathered granite stratum. When the soil cohesion increases from 20 kPa to 40 kPa, the vertical deformation of the tunnel decreases by 0.06 mm, and the horizontal deformation increases by 0.06 mm.

### 5.2 Construction influence of different internal friction angles

To study the influence law of soil internal friction angle on the mechanized construction of connecting passages, five groups of comparison working conditions were established by adjusting the internal friction angle of the completely weathered granite soil layer in the material model, with a gradient of 5° within the range of 20 ~ 40°, to investigate the influence of soil internal friction angle change on the mechanical response of connecting passage construction.

#### 5.2.1 Influence of different internal friction angles on ground settlement.

The influence of different internal friction angles of weathered granite stratum on ground settlement is shown in Fig 32.

**Fig 32:**
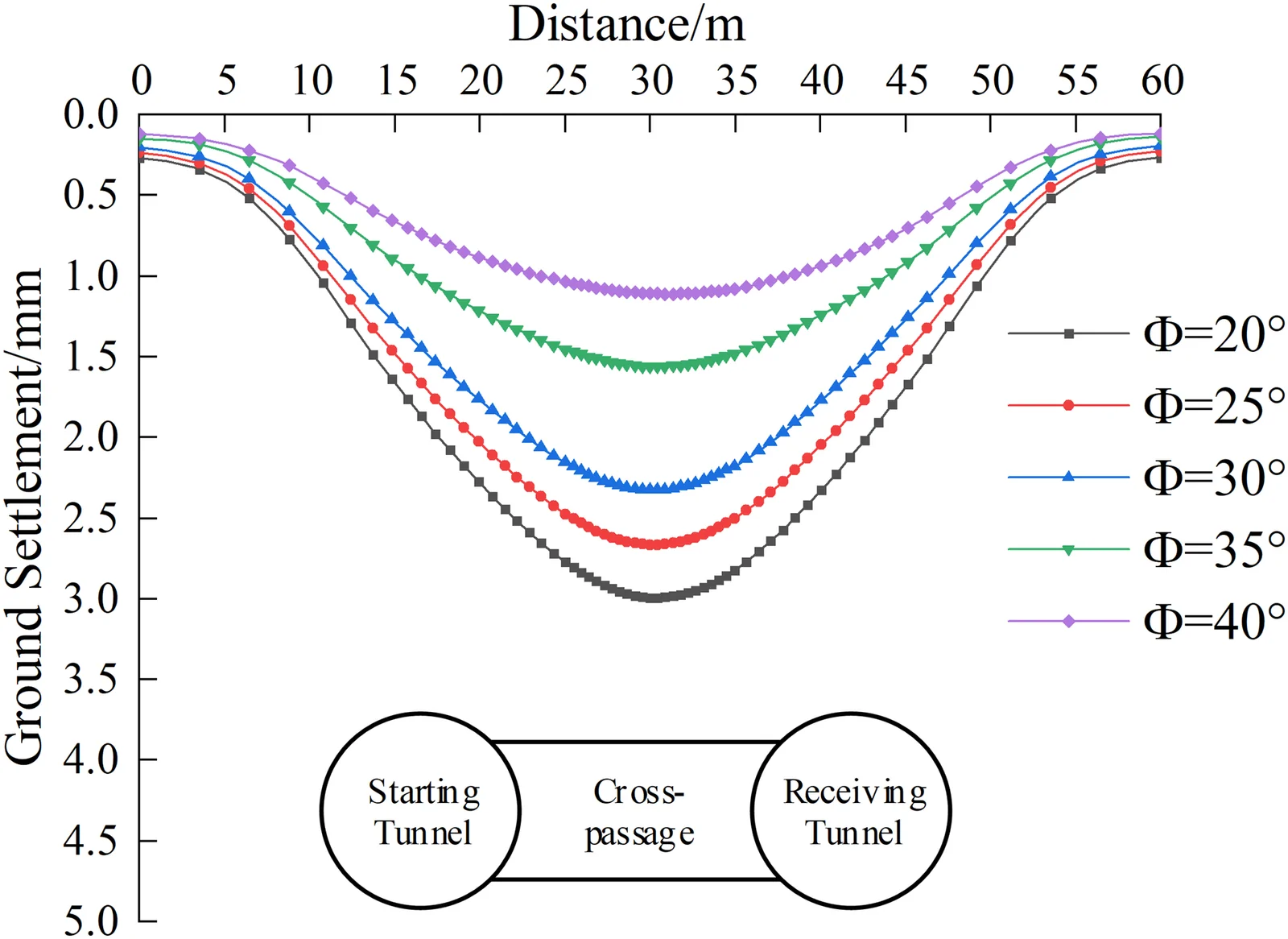
Ground settlement curves under different internal friction angle conditions.

It can be seen from Fig 32 that with the increase of the internal friction angle of the weathered granite stratum, the maximum ground settlement gradually decreases. When the internal friction angle of the weathered granite stratum increases to 40°, the settlement value decreases by 1.9 mm.

#### 5.2.2 Influence of different internal friction angles on deformation of main line tunnel.

The influence of different internal friction angles of weathered granite stratum on the deformation of the main line tunnel is shown in Fig 33.

**Fig 33:**
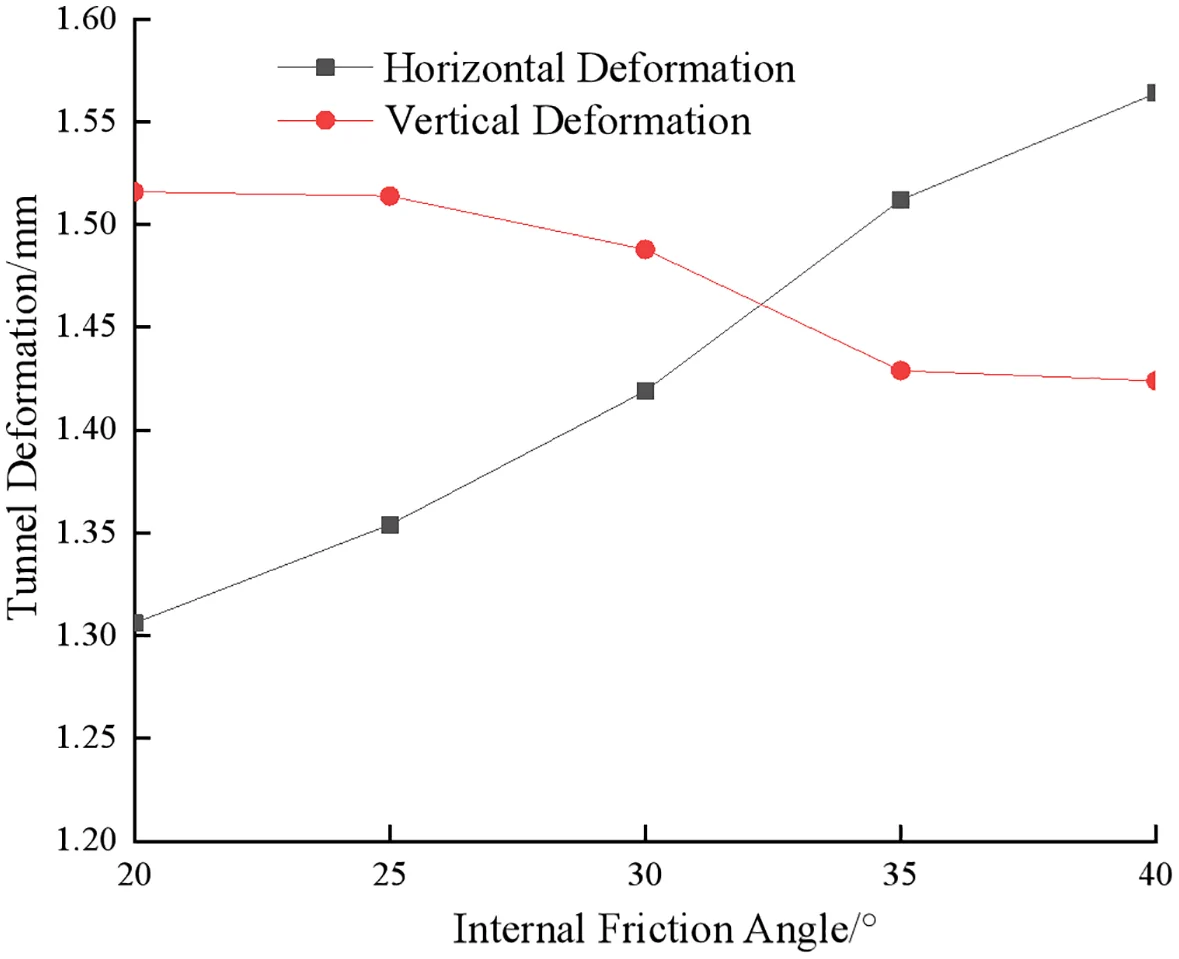
Deformation of the main line tunnel under different internal friction angle conditions.

It can be seen from Fig 33 that the maximum vertical deformation of the main line tunnel decreases with the increase of the internal friction angle of the weathered granite stratum, while the maximum horizontal deformation of the main line tunnel increases with the increase of the internal friction angle of the weathered granite stratum. When the soil internal friction angle increases from 20° to 40°, the vertical deformation of the tunnel decreases by 0.1 mm, and the horizontal deformation increases by 0.3 mm.

### 5.3 Construction influence of different clear distances between connecting passages

To study the influence law of different clear distances on the mechanized construction of connecting passages, models with different clear distance working conditions were established, and a total of 7 working conditions were set for analysis, in which the clear distance L was set to 13.0 m, 14.0 m, 15.0 m, 16.0 m, 17.0 m, 18.5 m, 20.0 m and 21.0 m respectively.

#### 5.3.1 Influence of different clear distances of connecting passages on ground settlement.

The influence of different clear distances on ground settlement is shown in Fig 34.

**Fig 34:**
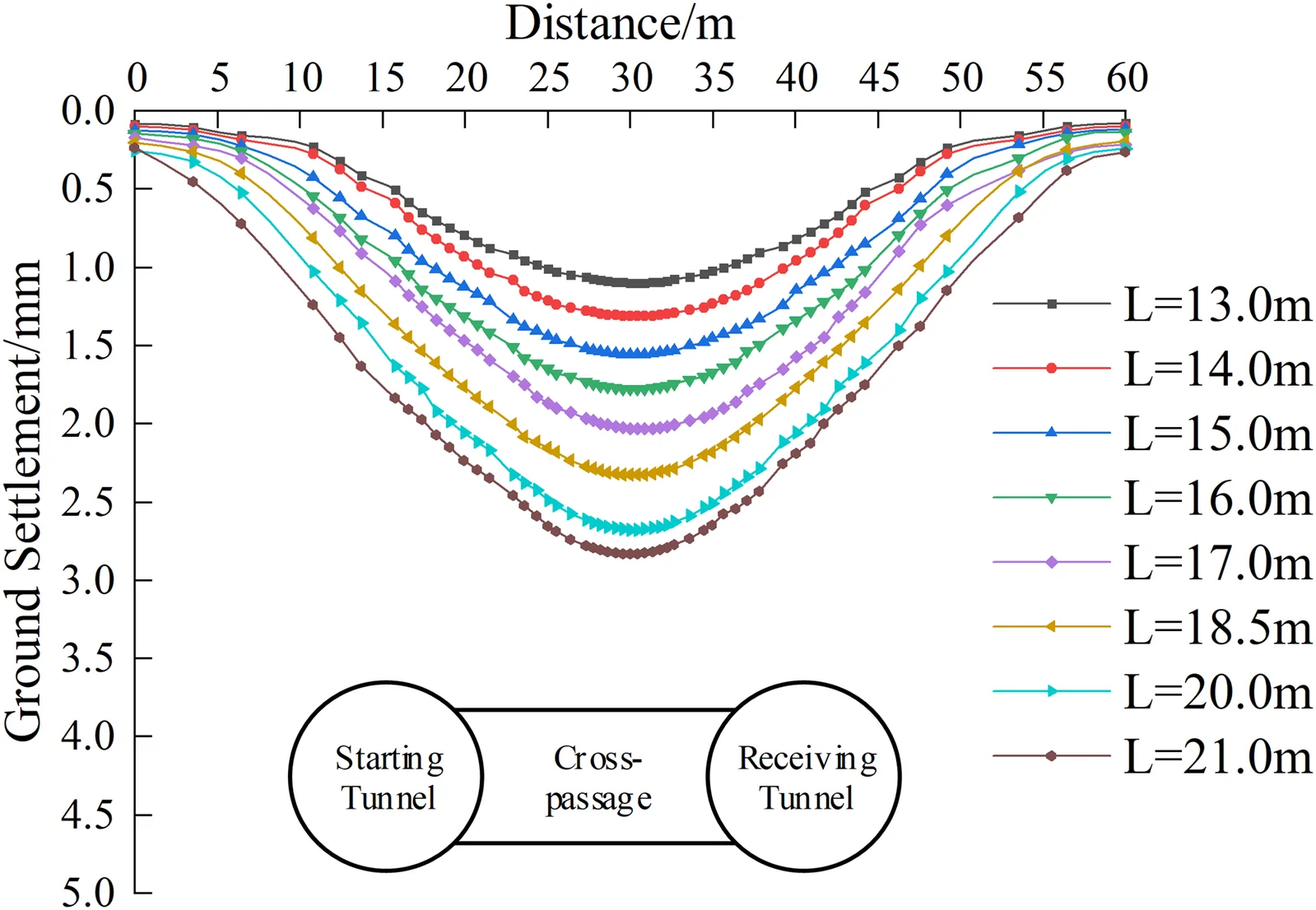
Ground settlement curves under different clear distance conditions.

It can be seen from Fig 34 that with the increase of the clear distance between connecting passages, the maximum ground settlement gradually increases. When the clear distance of the connecting passage increases from 13 m to 21 m, the maximum ground settlement increases by 1.7 mm.

#### 5.3.2 Influence of different clear distances of connecting passages on deformation of main line tunnel.

The influence of different clear distances on the deformation of the main line tunnel is shown in Fig 35.

**Fig 35:**
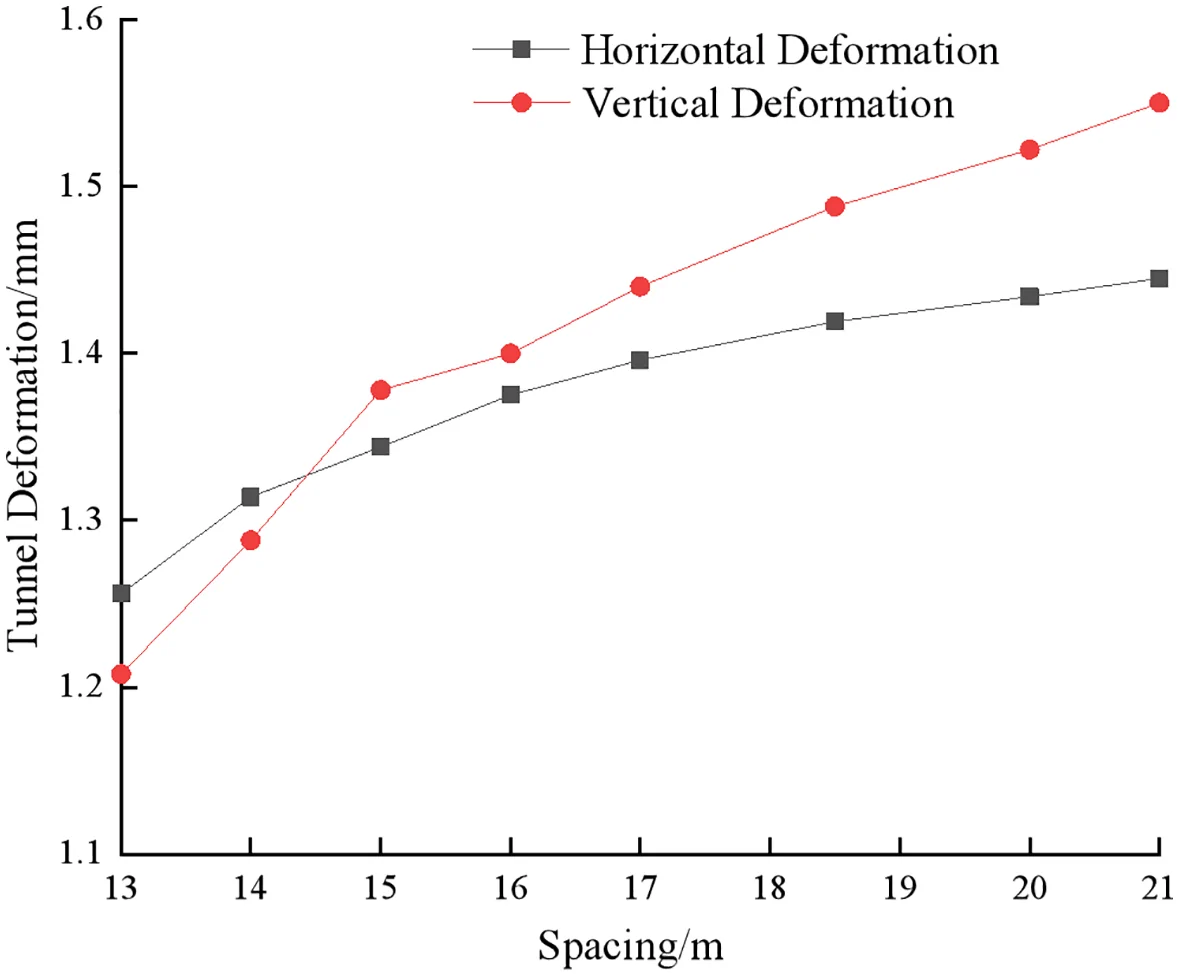
Deformation of the main line tunnel under different clear distance conditions.

It can be seen from Fig 35 that the maximum horizontal and vertical deformations of the main line tunnel increase with the increase of the clear distance of the connecting passage. When the clear distance of the connecting passage increases from 13 m to 21 m, the maximum horizontal and vertical deformations of the tunnel increase by 0.2 mm and 0.3 mm respectively.

### 5.4 Construction influence of different burial depths of connecting passages

To study the influence law of different burial depths on the mechanized construction of connecting passages, models with different burial depth working conditions were established, and a total of 8 working conditions were set for analysis, in which the burial depth H was set to 12 m, 14 m, 16 m, 18 m, 20 m, 22 m, 25 m and 27 m respectively.

#### 5.4.1 Influence of different burial depths of connecting passages on ground settlement.

The influence of different burial depths of the connecting passage on ground settlement is shown in Fig 36.

**Fig 36:**
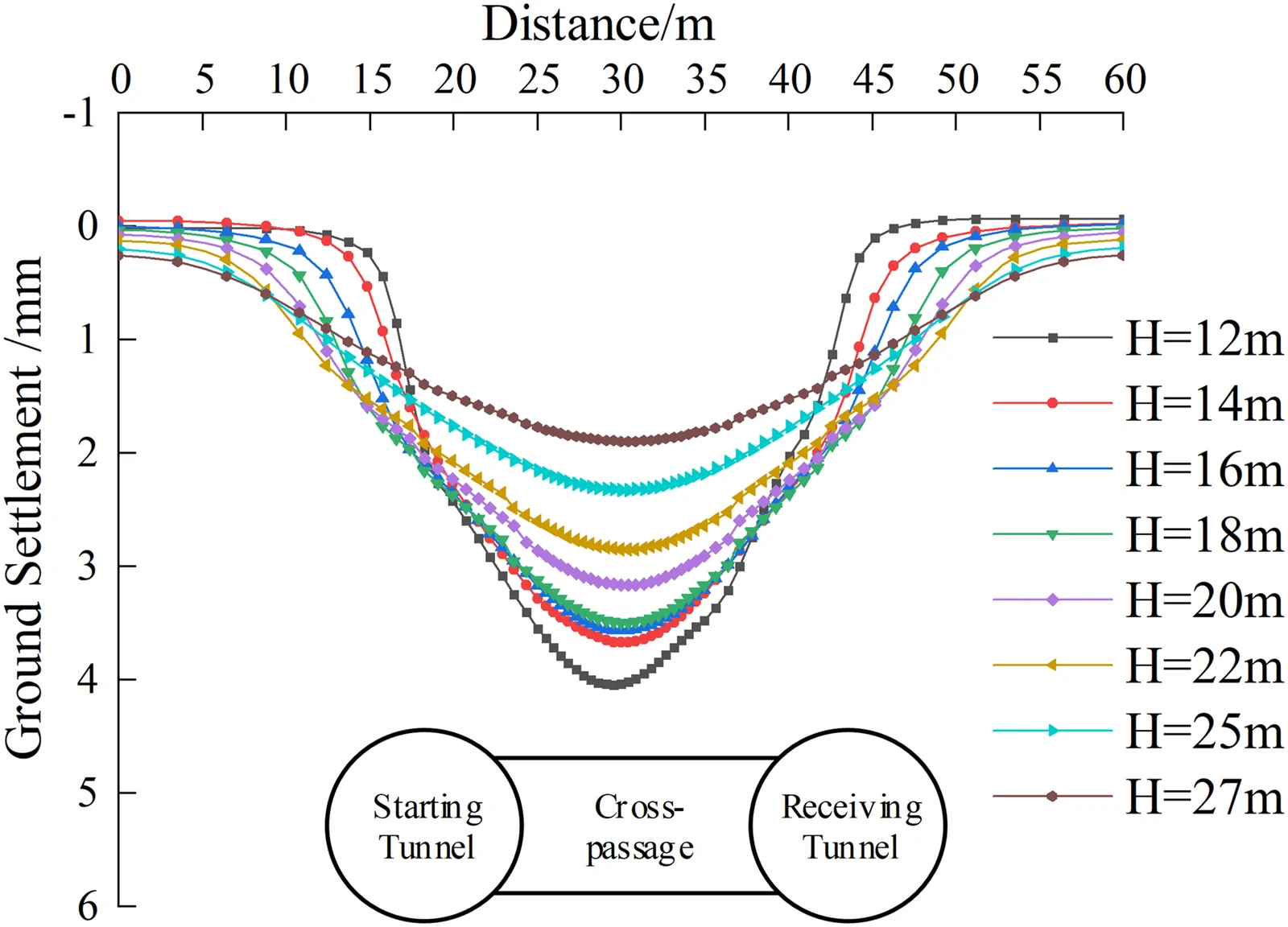
Ground settlement curves under different burial depth conditions.

It can be seen from Fig 36 that with the increase of burial depth, the settlement gradually decreases, but the settlement range expands. When the burial depth of the connecting passage increases to 27 m, the maximum value decreases by 2.1 mm.

#### 5.4.2 Influence of different burial depths of connecting passages on deformation of main line tunnel.

The influence of different burial depths on the deformation of the main line tunnel is shown in Fig 37.

**Fig 37:**
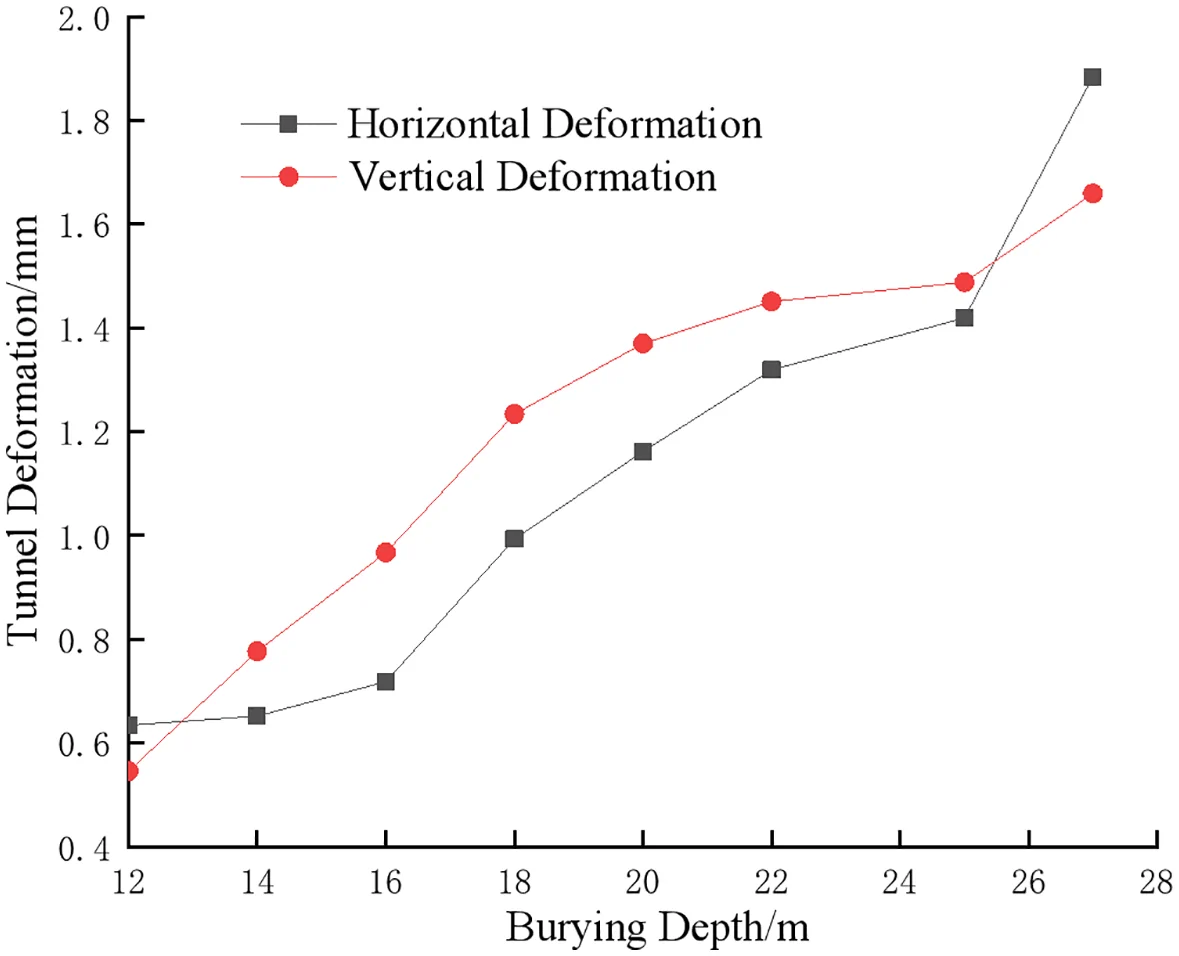
Deformation of the main line tunnel under different burial depth conditions.

It can be seen from Fig 37 that the deformation of the main line tunnel increases with the increase of the burial depth of the connecting passage, showing a positive correlation characteristic. When the burial depth of the connecting passage increases to 27 m, the maximum horizontal and vertical deformations of the main line tunnel increase by 1.3 mm and 1.1 mm respectively.

## 6 Conclusions

(1) The cutting ring at the launching end undergoes two sudden deformations during portal breaking: the deformation surges to the peak value at the first breaking, and then stabilizes; it increases significantly for the second time when the receiving end portal is broken. The maximum deformation of the cutting ring at the receiving end increases linearly with the advance of the pipe jacking machine before breaking, and tends to be stable after a sudden increase at the moment of breaking. The variation of bending moment shows that the bending moment at the launching end increases sharply during portal breaking, and then shows a trend of first rising and then slowly decreasing; the bending moment at the receiving end decreases slowly first and then rises during jacking, and surges to the maximum value during breaking.

(2) The initial growth rate of axial force at the receiving end is gentle, and the increase amplitude of axial force gradually increases with the increase of jacking distance, reflecting the asymmetric characteristic of load transfer between the two ends.

(3) After construction, a centrally symmetrical settlement trough is formed on the ground surface, with the maximum settlement of 2.3 mm located directly above the passage center. The deformation of the main tunnel presents directional difference: the peak value of Y-direction vertical deformation is 1.7 mm, and the X-direction horizontal deformation of 1.4 mm is significantly higher than the Z-direction deformation of 0.4 mm, reflecting the anisotropic response of stratum stress.

(4) The improvement of stratum strength (increase of cohesion and internal friction angle) can inhibit ground surface settlement, but aggravate the horizontal deformation of the main tunnel; the increase of the clear distance of the connecting passage will simultaneously increase ground surface settlement and tunnel deformation; the increase of burial depth reduces ground surface settlement and expands the settlement trough, but leads to the synchronous increase of horizontal and vertical deformation of the tunnel.
